# Supplementary material for: Improving gene set enrichment analysis (GSEA) by using regulation directionality
Source: Microbiol Spectr. 2024 Jan 31;12(3):e03456-23. doi: 10.1128/spectrum.03456-23 (PMC10913524; doi:10.1128/spectrum.03456-23)
Supplement: Supplemental text — GIN tool manual. [file spectrum.03456-23-s0001.pdf]

GINtool manual

Version: April 27, 2023

Contents

1. Introduction to the tool 2

2. Installation of GINtool 2

3. Overview of the tool 2

4. Preparing GINtool for analysis 6

5. Analysis of operons 8

6. Analysis of regulons 10

7. Analysis of functional categories 22

8. Overview of output sheets 35

9. Use of GINtool for transcriptome comparison 36

10. Calculation of MAD and average p-values 37

11. Making your own Bubble plot 37

1. Introduction to the tool

GINtool is an Excel plugin that is used for a comprehensive analysis of transcriptome data based on information on operons, regulons or functional categories.

This manual describes how to use GINtool. For each type of analysis, examples are shown.

2. Installation of GINtool

2.1. Preparation

Before installing GINtool, close Excel.

2.2. Installation

Click setup.exe to install GINtool.

> When clicking on setup.exe the following error message might appear: "*Customized functionality in this application will not work because the certificate used to sign the deployment manifest for GINtool or its location is not trusted. Contact your administrator for further assistance.*"
>
> In this case do the following:
>
> 1) Right click on setup.exe;
>
> 2) Select properties then the Digital Signatures tab;
>
> 3) Select "WIN-SMURF\frans" then click Details;
>
> 4) Click View Certificate and Click Install Certificate, go to Current user then Details and choose "Place all certificates in the following store";
>
> 5) Browse and select "Trusted Root Certification Authorities Store". Finally, finish and close the windows;
>
> 6) Click setup.exe again to install GINtool.

! In case Excel at some point starts to behave strangely, then uninstall GINtool and reinstall GINtool.

3. Overview of the tool

3.1 GINtool interface

GINtool is an Excel plugin that is added as a tab to the Excel toolbar after installation (see section 2). Figure 1 shows an overview of the tool. After clicking on the GINtool tab (Figure 1A, red box), the analysis bar appears (Figure 1B). From here you can move to the settings (main), select your data (select), activate filters (filter settings), choose the focus of your analysis (focus), create tables (tables), create plots (plots).

Via "show/hide settings" (Figure 1B, main) the settings bar appears (Figure 1C). The reference files on gene info, operons, regulons, categories and regulon info can be uploaded (reference files), cut-offs can be

determined (cut-offs) and sorting directions of the results can be chosen (sort direction). The columns of the reference files (see section 3.2) are linked to the tool by clicking on the reference file buttons and selecting the appropriate column names (column mapping, *e.g.* for regulons in Figure 1D, see section 4.1).

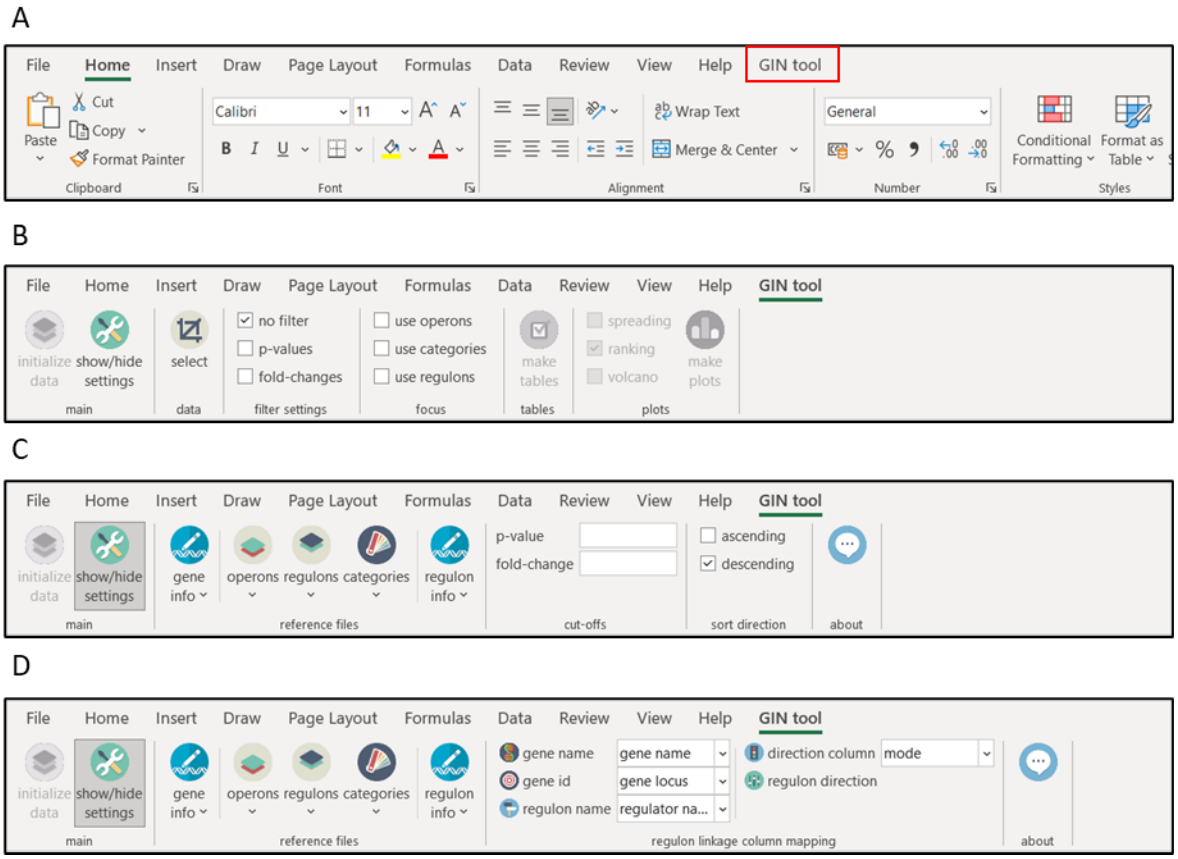

Figure 1: Overview of the GINtool plug-in. A) Location of GINtool tab in toolbar; B) Analysis bar; C) Settings bar; D) Regulon column mapping.

3.2 Overview of input reference files

GINtool works with 5 different information files: (i) A file containing information on the function of genes ("gene info"), (ii) a file indicating how genes are located in operons ("operons"), (iii) a file indicating to which regulon the gene belongs ("regulons"), (iv) a file indicating to which functional category the gene belongs ("categories"), and (v) a file containing information on the function of regulons ("regulon info"). These files can be imported either as txt files (*.csv) or Excel files (*.xlsx). The genes are linked to the information by either gene name or unique identifiers (ID).

The files shown in the examples are based on information obtained from *Subtiwiki*, a database for the model bacterium *Bacillus subtilis*. Figure 2 shows an overview of the different (.csv) reference files. Each reference file starts with a header for each of the columns in the file.

- The gene info reference file contains four columns: locus_tag, gene, function and description (Figure 2A). The gene ID, the gene name, gene function, and functional description are separated

by commas.

- The operon reference file contains one column: genes (Figure 2B). The operons are indicated as a list of gene names that are connected (e.g. yokI-yokJ-yokK-yokL). Single locus genes are indicated by single gene names (e.g. ywqG).
- The regulator reference file contains five columns: regulator locus, regulator name, mode (type of regulation), gene locus and gene name, separated by commas (Figure 2C). Importantly, GINtool does not use the regulator ID (*i.e.* regulator locus), because the regulator name is not always defined by a single regulator protein, for example the "stringent response" regulon.
- The category reference file contains three columns: category id, category and gene locus (Figure 2D). There are several layers of subcategories, and in total a depth of 5 categories with subcategories indicated by the identifier SW.x.x.x.x.x for the final subcategory. The category ID is followed by the description of the category/subcategory, and then the gene ID, separated by commas.
- The regulon info reference file contains three columns: regulon, regulon size (nr of genes) and function (Figure 2E). This information is not available in *Subtiwiki*, but was custom made based on the "gene info" information, where the function of the gene encoding the regulator is described. The information required is the name of the regulon, the number of genes in a regulon and the function of the regulon, separated by commas.

! The headers of the columns can be adjusted. When uploading the files to GINtool, the header names will be mapped to the corresponding elements (see section 4.1).

! GINtool only works when the "gene info column mapping" has been completed. It is also crucial to properly fill in the "regulon linkage column mapping", "category linkage column mappings" and/or "regulon info column mapping", when used.

! Gene info is mandatory for each analysis, in combination with at least the reference file(s) for either operons, regulons or categories.

A

	A	B	C	D	E	F	G
1	locus_tag,gene,function,description						
2	BSU_13700,clpE,protein degradation,"AAA unfoldase, ATPase subunit of the (						
3	BSU_30280,amyD,probably melibiose uptake,probably [SW|ABC transporter]						
4	BSU_30300,melA,melibiose utilization,alpha-galactosidase						
5	BSU_30290,amyC,probably melibiose uptake,probably [SW|ABC transporter]						
6	BSU_30270,msmE,probably melibiose uptake,probably ABC transporter for m						
7	BSU_37350,sboA,antimicrobial peptide,subtilosin A						
8	BSU_30260,msmR,regulation of melibiose utilization,transcriptional regulato						
9	BSU_39760,iolA,myo-inositol catabolism,methylmalonate-semialdehyde deh						
10	BSU_06230,iolT,myo-inositol uptake,major transporter for inositol						

B

	A	B	C	D	E	F	G
1	genes						
2	ywqG						
3	yokI-yokJ-yokK-yokL						
4	mbl						
5	coaX-yacC-yacD-cysK						
6	htrC						
7	rpmGA						
8	scoC						
9	pepF						
10	pucJ-pucK-pucL-pucM						

C

	A	B	C	D	E	F	G
1	regulator locus,regulator name,mode,gene locus,gene name						
2	BSU_36420,spoIIID,activation,BSU_06900,cotJB						
3	BSU_36420,spoIIID,activation,BSU_06890,cotJA						
4	BSU_36420,spoIIID,activation,BSU_06910,cotJC						
5	BSU_36420,spoIIID,activation,BSU_06920,yesJ						
6	BSU_36420,spoIIID,activation,BSU_06930,yesK						
7	BSU_15320,sigE,sigma factor,BSU_06900,cotJB						
8	BSU_15320,sigE,sigma factor,BSU_06890,cotJA						
9	BSU_15320,sigE,sigma factor,BSU_06910,cotJC						
10	BSU_15320,sigE,sigma factor,BSU_06920,yesJ						

D

	A	B	C	D	E	F	G
1	category id,category,gene locus						
2	SW.6.9.1,6S RNA,BSU_misc_RNA_32,						
3	SW.6.9.1,6S RNA,BSU_misc_RNA_41,						
4	SW.3.1.5.2,A/P endonucleases,BSU_40880,						
5	SW.3.1.5.2,A/P endonucleases,BSU_25130,						
6	SW.3.1.5.2,A/P endonucleases,BSU_22340,						
7	SW.2.6.5.4,ABC transporters for the uptake of iron/ siderophores,BSU_3329(						
8	SW.2.6.5.4,ABC transporters for the uptake of iron/ siderophores,BSU_3331(						
9	SW.2.6.5.4,ABC transporters for the uptake of iron/ siderophores,BSU_0163(						
10	SW.2.6.5.4,ABC transporters for the uptake of iron/ siderophores,BSU_0162(						

E

	A	B	C	D	E	F	G
1	regulon,regulon size (nr of genes),function						
2	Abh,22,regulation of gene expression during the transition from growth to sta						
3	a-box,1,"termination/antitermination pbuE (putative hypoxanthine exporter,						
4	AbrB,271,regulation of gene expression during the transition from growth to :						
5	AcoR,4,regulation of acetoin utilization						
6	AdaA,3,adaptive response to alkylative DNA damage						
7	AdeR,1,control of L-alanine dehydrogenase expression (alanine utilization)						
8	AdhR,2,regulation of the protective response to formaldehyde and methylgly						
9	AhrC,16,transcriptional regulator of arginine metabolic genes						
10	AlsR,2,regulation of acetoin synthesis						

Figure 2: Overview of input files. A) Gene info; B) Operons; C) Regulons; D) Categories; E) Regulon info.

3.3 Overview of input dataset

Datasets that are analyzed with GINtool are the result of a differential expression analysis of your transcriptomics data. The file includes a column with gene names, fold-changes and p-values (or a related statistical classifier). Figure 3 shows an example. The fold-changes can be log2 transformed, but do not have to. Importantly, a minus sign (-) is required to indicate downregulation.

! Importantly, remove non-numerical values (like “NA”) for fold-change and p-values.

	A	B	C	D
1	**locus**	**log2FC**	**p-val**	
2	BSU_11549	2.002516245	0.096155	
3	BSU_21030	1.62902704	0.22792	
4	BSU_14629	1.601961295	0.002607	
5	BSU_36720	1.504901617	0.152471	
6	BSU_20300	1.504410801	0.181841	
7	BSU_06319	1.503871939	0.177237	
8	BSU_27809	1.476434051	0.218652	
9	BSU_06120	1.440394618	0.039961	
10	BSU_20620	1.409932161	0.183179	
11	BSU_32090	1.395148599	0.019007	
12	BSU_07190	1.392904273	0.180525	

Figure 3: Example of transcriptomics data. The data contains three columns: a column containing gene IDs, a column with log2-transformed fold-changes and a column with p-values.

! Gene IDs in the dataset need to match the gene IDs in the reference files. For example, BSU11549 is not the same as BSU_11549.

4. Preparing GINtool for analysis

*First prepare the transcriptomics data and reference files according to **section 3**.*

4.1 First use: upload files

Open your transcriptomics data in Excel and open GINtool via the tab in the toolbar (Figure 1A). Click "show/hide settings" to open the settings. Upload the reference files for gene info and the analysis you want to perform (*i.e.* operon, regulon or functional categories) by clicking on the appropriate buttons and selecting the files (Figure 4A). After uploading each file, link the column names to the correct element ("show/hide column mapping", Figure 1D).

In case of regulon information, also determine regulation directions (Figure 4B). The left box called "undefined" lists all definitions found under the "direction column" which is labelled "mode" in the example. For a complete regulon analysis it is necessary to separate the different types of regulation into activation and repression by moving them either into the "up-regulated" or "down-regulated" box. When the type of regulation is unknown it can be left in the "undefined" box. Of note, we consider "sigma factors" activators. This information will be maintained in Excel for use in next sessions, as long as you use the same regulon file (see 4.3).

! Gene info is mandatory for each analysis, in combination with at least the reference files for either operons, regulons or categories.

4.2 Select transcriptomics data

After uploading and mapping the reference data, hide the settings by clicking "show/hide settings". The "select" button should now be enabled. If not, click "initialize data". Click the "select" button, select the appropriate columns of your transcriptomics data (Figure 4C) and click "OK". The analysis bar will now be

enabled. Of note, the program will need some time, about a minute, to calculate enrichment scores for average p-value calculations.

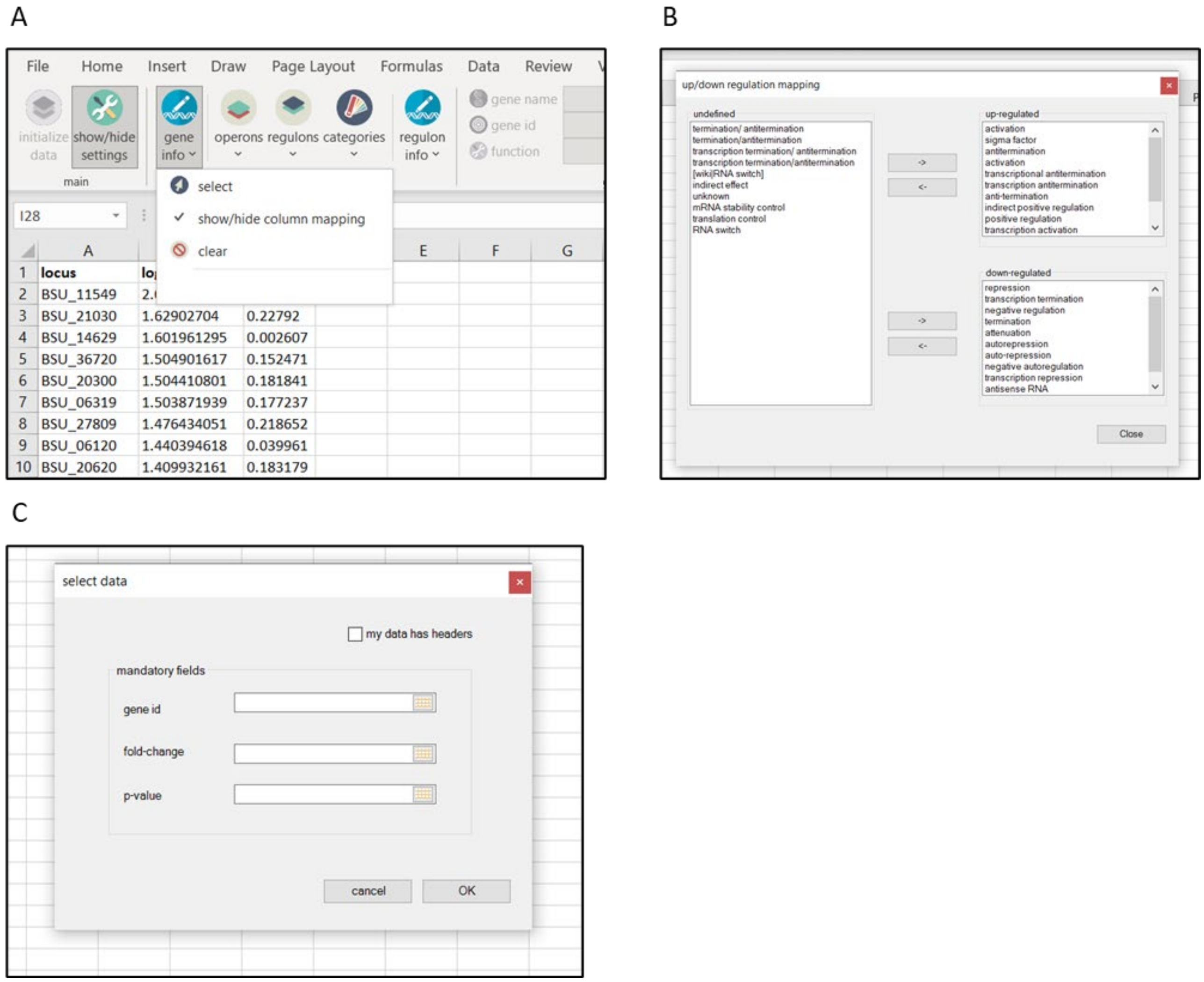

Figure 4: Uploading reference files and selecting your data. A) Selection of reference file for gene info; B) Selection of Up/down regulation mapping of regulons; C) Selecting of columns of transcriptomics data.

<u>4.3 Subsequent use: continue using GINtool after closing Excel</u>

After restarting Excel, you may need to repeat some of the steps described in 4.1 and 4.2. If the analysis buttons are disabled, you need to select the data columns again. If this is not possible, check if the reference files are still coupled by clicking on the arrow buttons beneath the reference file buttons. If the files are still coupled, click "<u>initialize data</u>" button in order to reload the files. If the files are not coupled, upload the files again, map the correct columns and click "<u>initialize data</u>". Now you are again able to select your data and continue your analysis.

5. Analysis of operons

*First follow the steps described in **section 4**.*

5.1 Fold-changes of genes in operons

To display fold-changes in operons, tick "use operons" in the "focus" window (Figure 5A, red box) and click "make tables" in the "tables" window. This will create a new sheet called "Mapped" (Figure 5B). In "Mapped", each row represents the information for one gene. Of note, the fold-changes in operon data begins in column K. To make this more clear, we used the Excel option "Conditional formatting" to highlight fold-change differences using colors. Column D shows the gene and column G the operon to which the gene belongs. The structure of the operon is indicated in column J, and column I shows the number of genes in the operon. Figure 5B, row 234, gives a clear example of an operon that is not regulated like an operon, since the two genes in the *yxaJ* operon shows very different fold-changes. Sometimes there are different transcriptional start sites in an operon, *e.g.* this is shown in row 233 and 238 in Figure 5B. In this case column H gives the value 2 for the number of operons. Cell K236 is empty, because for some reason there is no proper fold-change data for the gene *yfnH*.

In the example above, the tick box "no filter" in the window "filter settings" is activated (Figure 5A), thus all transcriptomics data is used in this analysis. If you wish to prefilter genes, you can specify cut-off values for either the fold-change or p-value of the genes that are included in the analysis. Click "show/hide settings" to open the settings bar. The desired cut-offs can be determined in the cut-offs window (Figure 5C, red box). By default, no cut-offs are activated. To use a cut-off, click "show/hide settings" and tick "fold-changes" in the "filter settings" window in the analysis bar (Figure 5D, red box). The table can be created as described above. For example, the FC cut-off can be changed to 2. After filtering the genes based on a fold-change cut-off of 2, only 32 operons remain (Figure 5E). The same procedure can be followed to select genes based on p-values, or based on both cut-offs, by ticking the appropriate boxes in "filter settings" (Figure 5D).

! If the fold-changes in the data set are log2 transformed, then the FC cut-off is also interpreted as such.

! The fold-change cut-offs are absolute values, *i.e.* fold 2 can be both 2-fold downregulation and 2-fold upregulation.

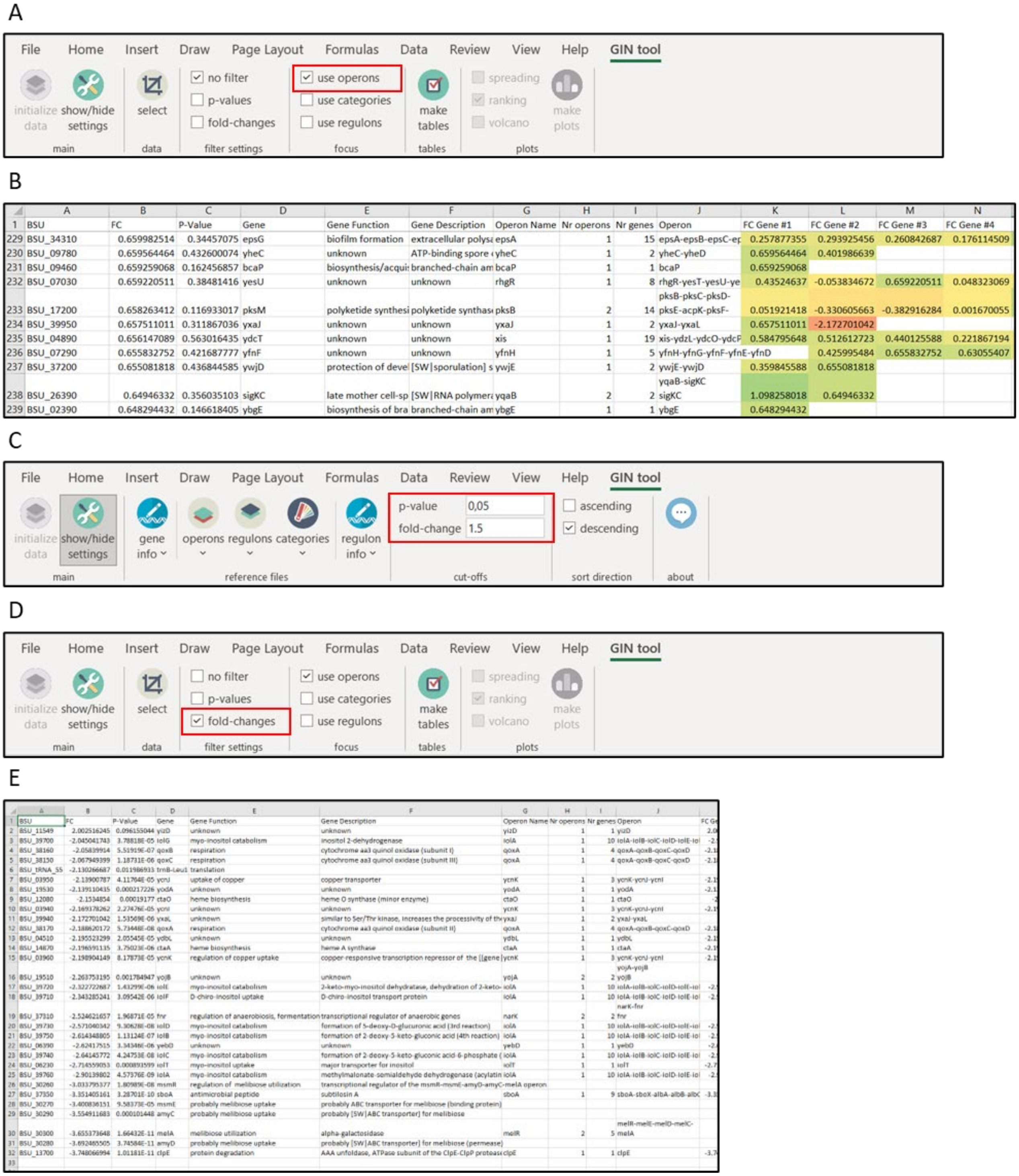

Figure 5: Analysis of operons. A) Selection of operons as focus of the analysis; B) Snapshot of “Mapped”. Fold-changes are colored by conditional formatting; C) Cut-off settings; D) Activating fold-change filter; E) Overview of “Mapped” after filtering based on a fold-change of 2.

6. Analysis of regulons

*First follow the steps described in **section 4**.*

6.1 Visual analysis of regulons using spread plots

GINtool gives the option to rank regulons based on the average fold-change of all genes in a regulon. The distribution of fold-changes of genes of regulons can be visualized in a spread plot. Start by ticking "use regulons" as "focus" and "spreading" for "plots" (Figure 6A). Click "make plots", the "Select categories/regulons" window will pop up (Figure 6B).

By clicking on "Regulons" in the left window "Available categories/regulons", all regulons become visible. Regulons can be selected and moved to the right window "Selection" by clicking on the single arrow button. It is also possible to select all regulons by clicking the double arrow. To produce the spread plot, click "Ok". This will create a new sheet called "RegSpreadPlot", displaying the fold-changes of all genes in the selected regulons. The regulons are ranked based on the average fold-change (Figure 6C). The x-axis indicates the fold-change. In the example all regulons were selected.

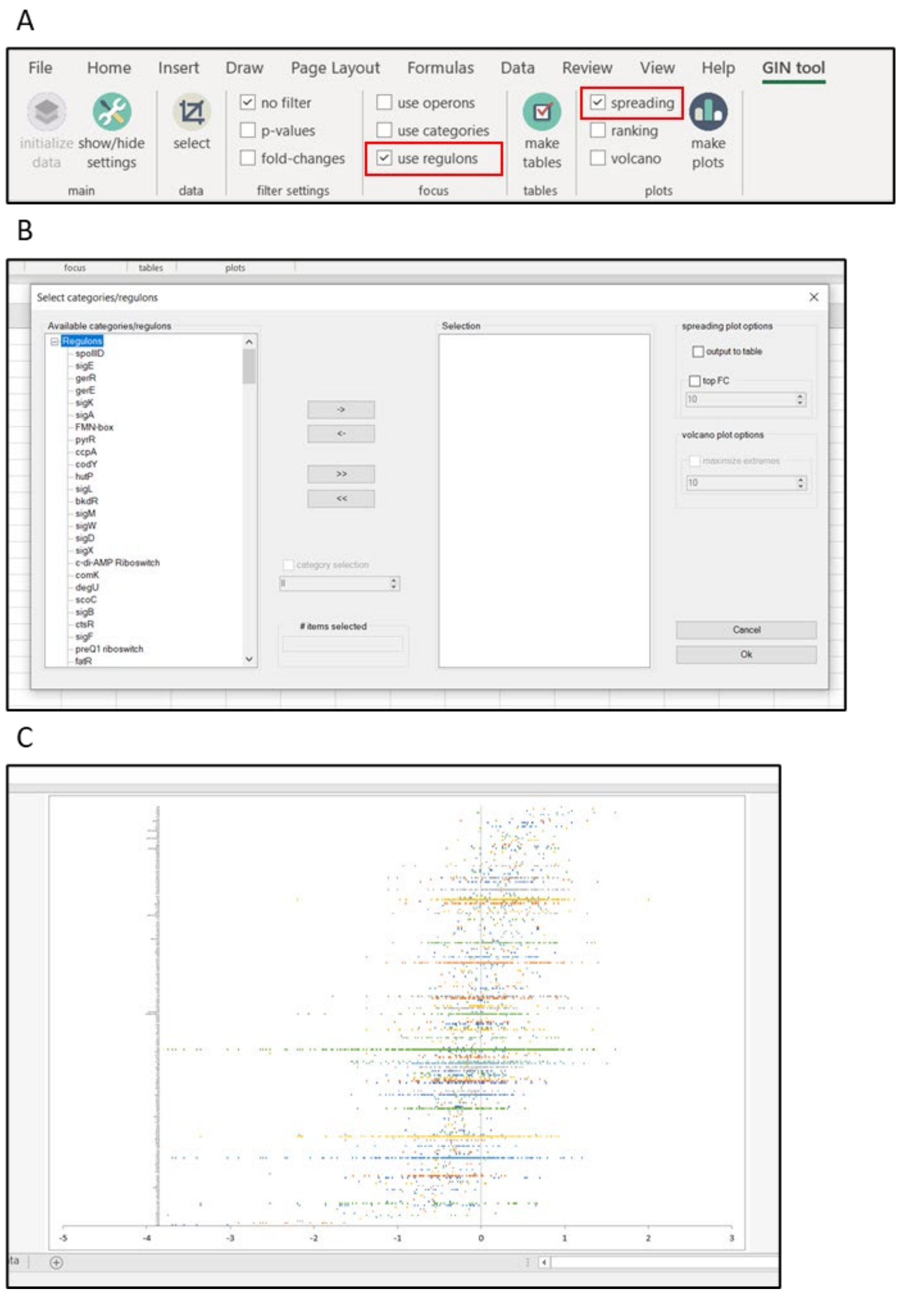

Figure 6: Creating a spread plot of all regulons. A) Selection of focus and type of plot; B) Selection of regulons of interest; C) Spread plot "RegSpreadPlot" with information of fold-changes of all genes in all regulons, in descending order.

Often only the most up- or downregulated regulons are interesting. To show these, go back to "make plots" and tick the "top FC" (fold-change) box and set the number of most strongly regulated regulons to be displayed, *e.g.* 20 (Figure 7A, red box). By clicking on "Ok" a new sheet is created called "RegSpreadPlotTop20FC", displaying the 20 most affected regulons (Figure 7B).

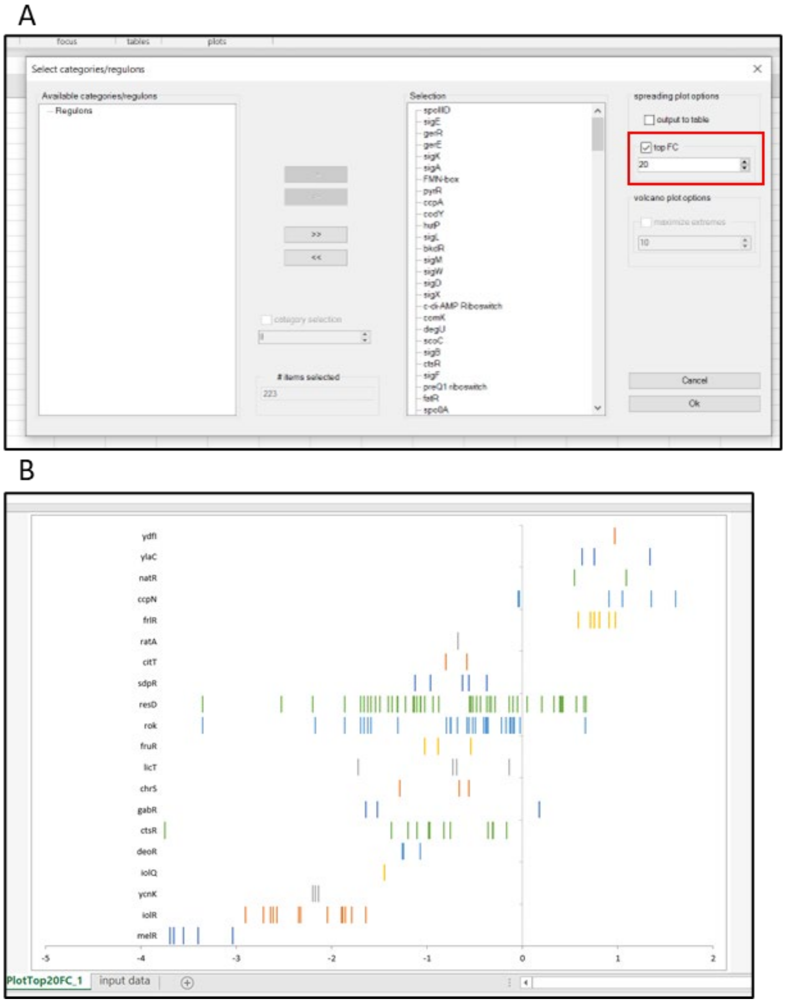

Figure 7: Creating a spread plot of top 20 affected regulons based on average fold-changes. A) Selection of spreading plot options; B) Plot of top 20 most affected regulons based on average FC "RegSpreadPlotTop20FC".

As default the most upregulated regulons are displayed on top. This order can be reversed. For this, click "show/hide settings" and the "sort direction" window becomes visible (Figure 8A, red box). Tick "ascending", click on "show/hide settings" and "make plots", select the regulons of interest, if necessary also select the best scoring regulons using the FC or p-value selection, and click "Ok". Figure 8B shows the reversed result of Figure 6C.

A

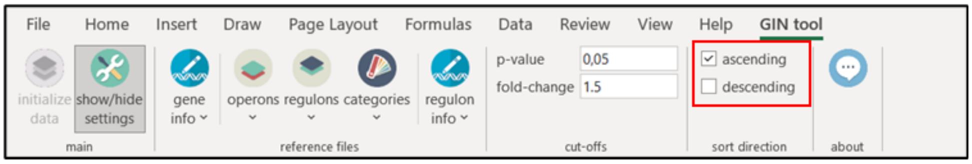

B

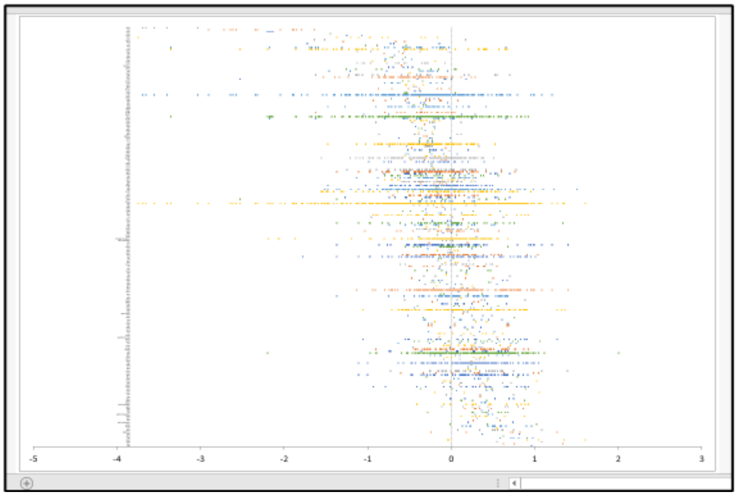

Figure 8: Creating a spread plot of all regulons in ascending order. A) In settings bar, ticking direction in "sort direction"; B) Spread plot "RegSpreadPlot" with information of fold-changes of all genes in all regulons, in ascending order.

It can be useful to know what genes are part of the selected regulons and what their fold-change and p-values are. To obtain this information click again on "make plots" but this time tick the "output to table" box (Figure 9A, red box). In the example the 10 strongest up or down regulated regulons have been selected. After clicking "Ok" a sheet called "RegSpreadTabTop10FC" is created, listing the selected regulons with the genes that are part of the regulon and the related fold-change (FC) (Figure 9B). Besides, a sheet with the corresponding spread plot called "RegSpreadPlotTop10FC" is created.

A

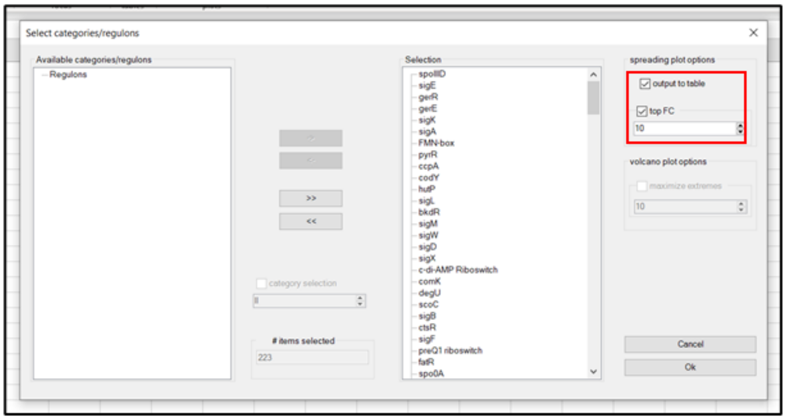

B

	A	B	C	D
1	Regulon	Gene	FC	p-value
2	ydfI	ydfJ	0.964749	0.145385
3	ylaC	ylaB	1.3398	0.206763
4	ylaC	ylaC	0.752457	0.312897
5	ylaC	ylaA	0.623017	0.346254
6	chrS	chrS	-0.5587	0.223859
7	chrS	ywrB	-0.6636	0.176841
8	chrS	ywrA	-1.28899	0.013897
9	gabR	gabR	0.173587	0.693414
10	gabR	gabD	-1.51829	6.2E-05
11	gabR	gabT	-1.64061	0.000586
12	ctsR	ysxC	-0.16648	0.635803
13	ctsR	lonA	-0.31004	0.341778
14	ctsR	clpX	-0.31201	0.385095
15	ctsR	trxA	-0.35974	0.321492
16	ctsR	disA	-0.7549	0.03259
17	ctsR	radA	-0.82336	0.025296

Figure 9B: Generation of table with fold-change and p-value of genes in selected regulons. A) Selection of settings in "Select categories/regulons"; B) Resulting table in sheet "RegSpreadTabTop10FC".

An alternative way to select regulons is by specifying cut-off values for either the fold-change or p-value of the genes that are included in the analysis. Click "show/hide settings" to open the settings bar. The desired cut-offs can be determined in the cut-offs window (Figure 10A, red box). By default, no cut-offs are activated. To use a cut-off, click "show/hide settings" and tick "fold-changes" in the "filter settings" window in the analysis bar (Figure 10B, red box). The plots and tables can be created as described above. For example, the FC cut-off can be changed to 2. After filtering the genes based on a fold-change cut-off of 2, only 15 regulons remain (Figure 10C). The same procedure can be followed to select genes based on p-values, or based on both cut-offs, by ticking the appropriate boxes in "filter settings" (Figure 10B).

! If the fold-changes in the data set are log2 transformed, then the FC cut-off is also interpreted as such.

! The fold-change cut-offs are absolute values, *i.e.* fold 2 can be both 2-fold downregulation and 2-fold upregulation.

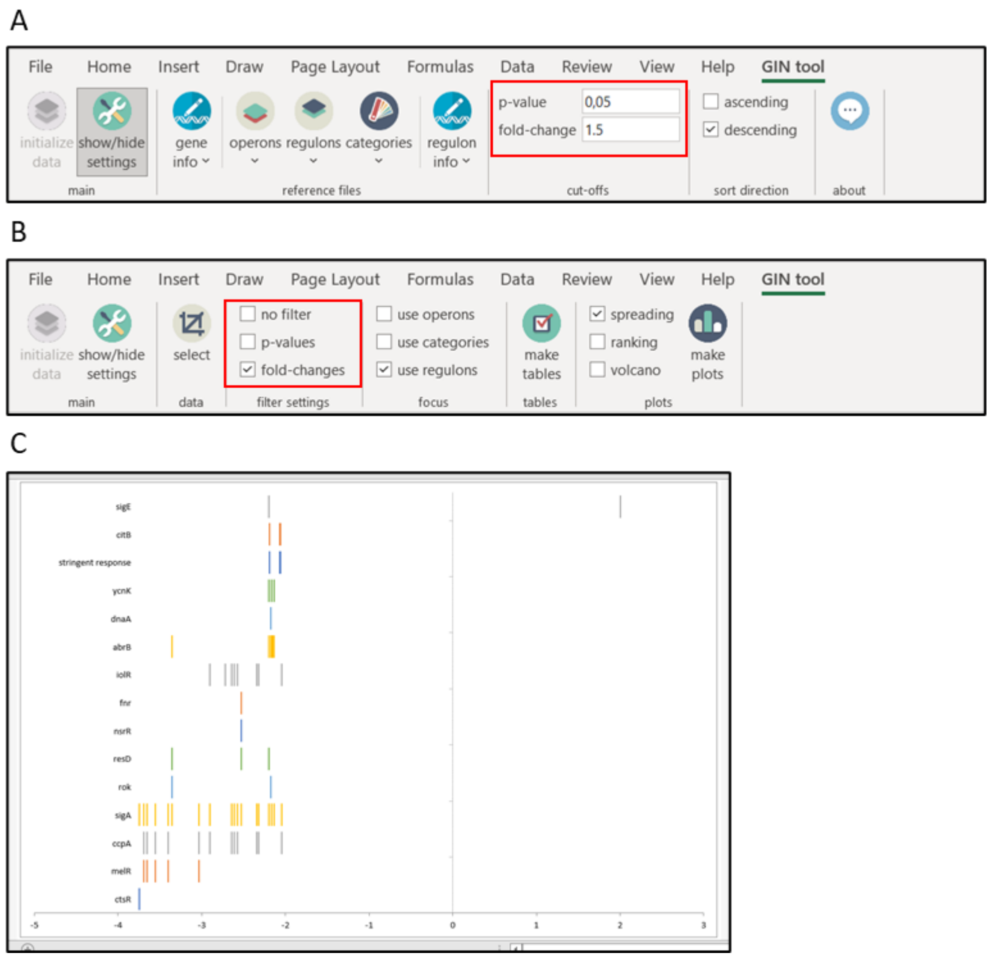

Figure 10: Creating spread plots of regulons after filtering genes. A) Cut-off values can be specified in the "cut-offs" window in the settings bar; B) Cut-offs can be activated by ticking the appropriate boxes in "filter settings" in the analysis bar; C) Example of "RegSpreadPlot" after filtering genes below a fold-change of 2.

6.2 Visual analysis of regulons using bubble plots

The large number of regulons can make it difficult to visually determine how relevant they are. To facilitate this, GINtool can make bubble plots based on the average fold-change of the genes in a regulon (x-axis) and the spreading of fold-changes of genes, defined as the mean absolute deviation (MAD, see 10) of the fold-changes (y-axis). Regulons that show a robust regulation should have fold-changes that are close together, resulting in lower MAD values.

To make these bubble plots, tick "use regulons" in the "focus" window and "ranking" in the "plots" window (Figure 11A, red boxes). Click "make plots" and the "select categories/regulons" window will pop up. Select the relevant regulons as described above (section 6.1). Of note, for this analysis it is not possible to select regulons with e.g. highest fold-change, however, fold-change and p-value cut-offs for genes can be activated (Figure 10A). In the example below, no filters are active and all regulons are selected.

After selecting regulons and clicking "Ok", four sheets are created: "RegRankPlot", "RegRankPlotBest_v1",

“RegRankPlotBest_v2” and “RegRankTable”. “RegRankPlotBest_v1” and “RegRankPlotBest_v2” will be discussed in section 6.4. An example of “RegRankPlot” is shown in Figure 11B.

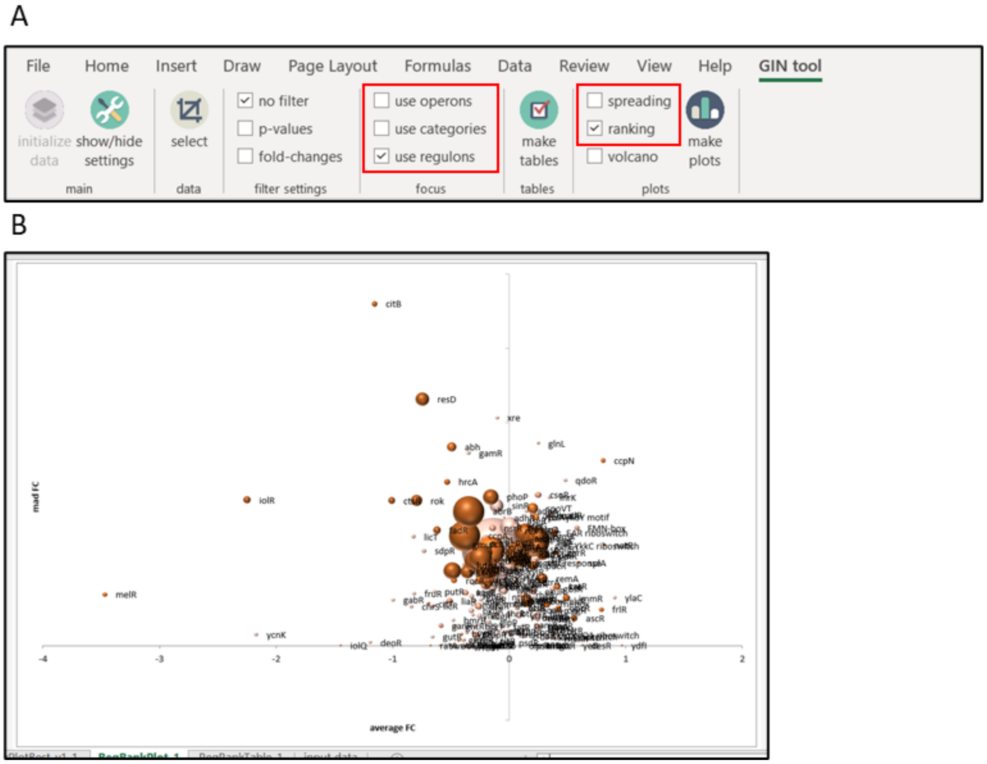

Figure 11: Creating a bubble plot of the average regulation of all regulons. A) Selection of correct settings for creating bubble plots of regulons; B) Example bubble plot “RegRankPlot” of all regulons. Color intensity corresponds to ranking in average p-values. Bubble size corresponds to the number of genes in a regulon.

The use of a bubble plot provides the possibility to also display the size of regulons, *i.e.* the number of genes that are part of a regulon. The example in Figure 11B displays bubbles with different color intensities. The color intensity is related to the average p-value of the genes in the regulon.

The regulons are divided into 5 fractions based on average p-values: the 10 % regulons with the lowest p-values, then the 10-20 %, 20-33 % and 33-50 % fractions, and finally a fraction containing half of all regulons (50 %) with the highest p-values. This classification enables the removal of regulons with high average p-values by using the Excel “Chart filter” tool, which is activated by clicking on the graph, as shown in Figure 12. The bubbles are larger than in Figure 11B, because Excel works with relative measures for bubble scaling. The scale can be adjusted using the “Format data series” tool that becomes available by right-clicking on a bubble (Figure 12B). In this example, the scale is reduced to 25. Of course, the plots can be converted in other type of graphs and adjusted using standard Excel tools, and the graphs can be exported for furter editing, which can be useful for repositionion regulon names or changing the size or color of bubbles.

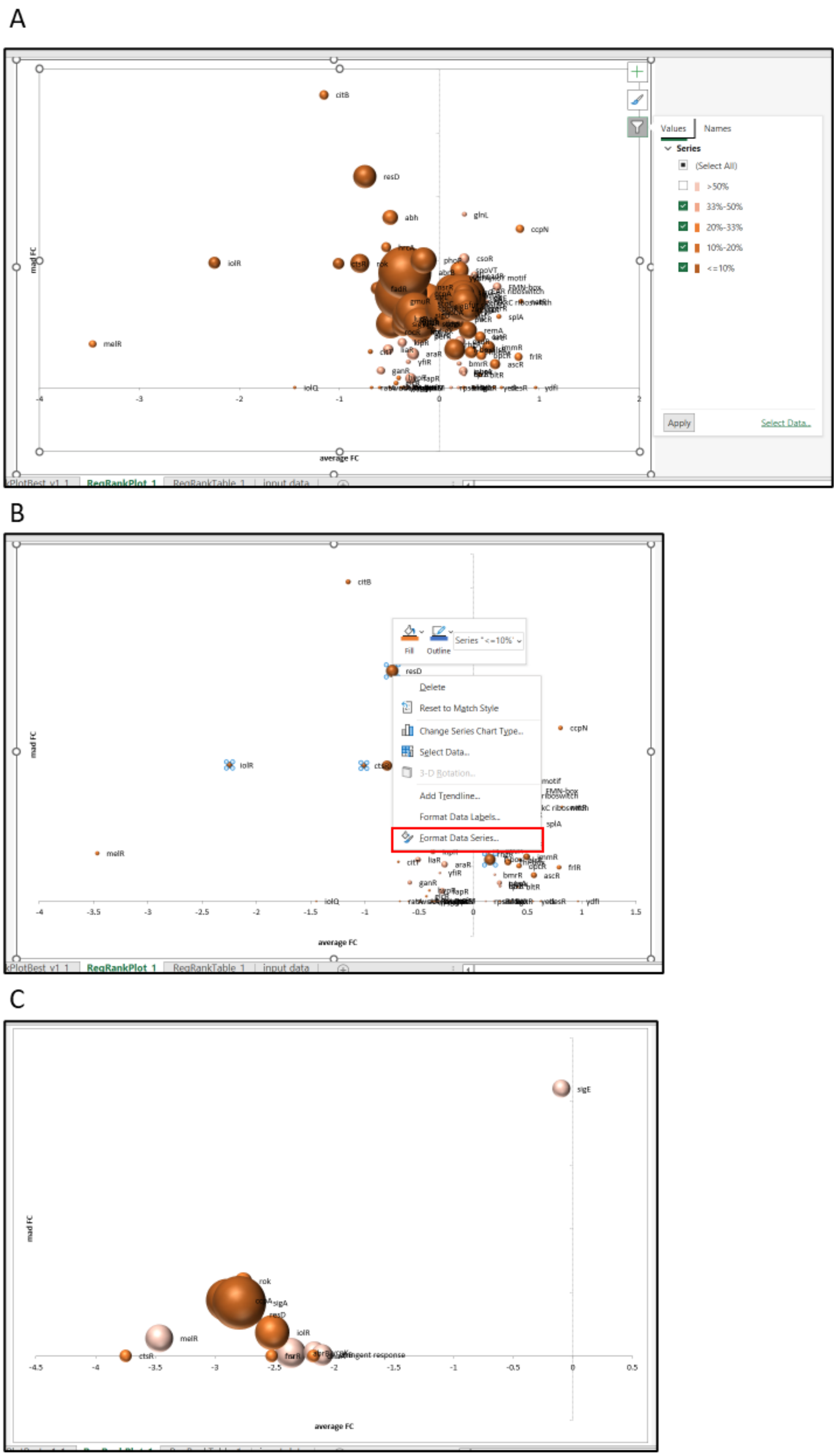

Figure 12: Creating bubble plots of regulons based on preferences. A) Selecting bubbles based on average p-value of genes in a regulon; B) Rescaling bubble sizes; C) Bubble plot of regulons after filtering genes with FC cut-off of 2.

It is possible to display the bubble plots of regulons after filtering the genes in the regulons based on fold-change and p-value (Section 6.1, Figure 10A). When a fold-change cut-off of 2 is active, fewer regulons remain and distribution of colors is now based on the ranking of these 15 regulons (Figure 12C).

6.3 Regulon evaluation using tables

The average fold-change, MAD- and p-values can also be listed in a table. To do this, click on the "make tables" tab in the "tables" window (Figure 13A, red box). This creates two sheets: "Mapping_details" and "Mapped". "Mapped" will be discussed in section 6.4. The "Mapping_details" sheet contains four different tables (Figure 13B). The first table "Without regulatory directionality" shows the number of genes, average fold-change, MAD and average p-value for each regulon. The table enables a quick ranking according to any of these parameters, by selecting the headers and using the Excel option "Filter" in the "Sort & Filter" tab in the Home tab (Figure 13C, red box). Of note, when making bubble plots there is also a table created "RegRankTable". This table is the same as the "Mapping_details" table, but then showing only the regulons that were selected.

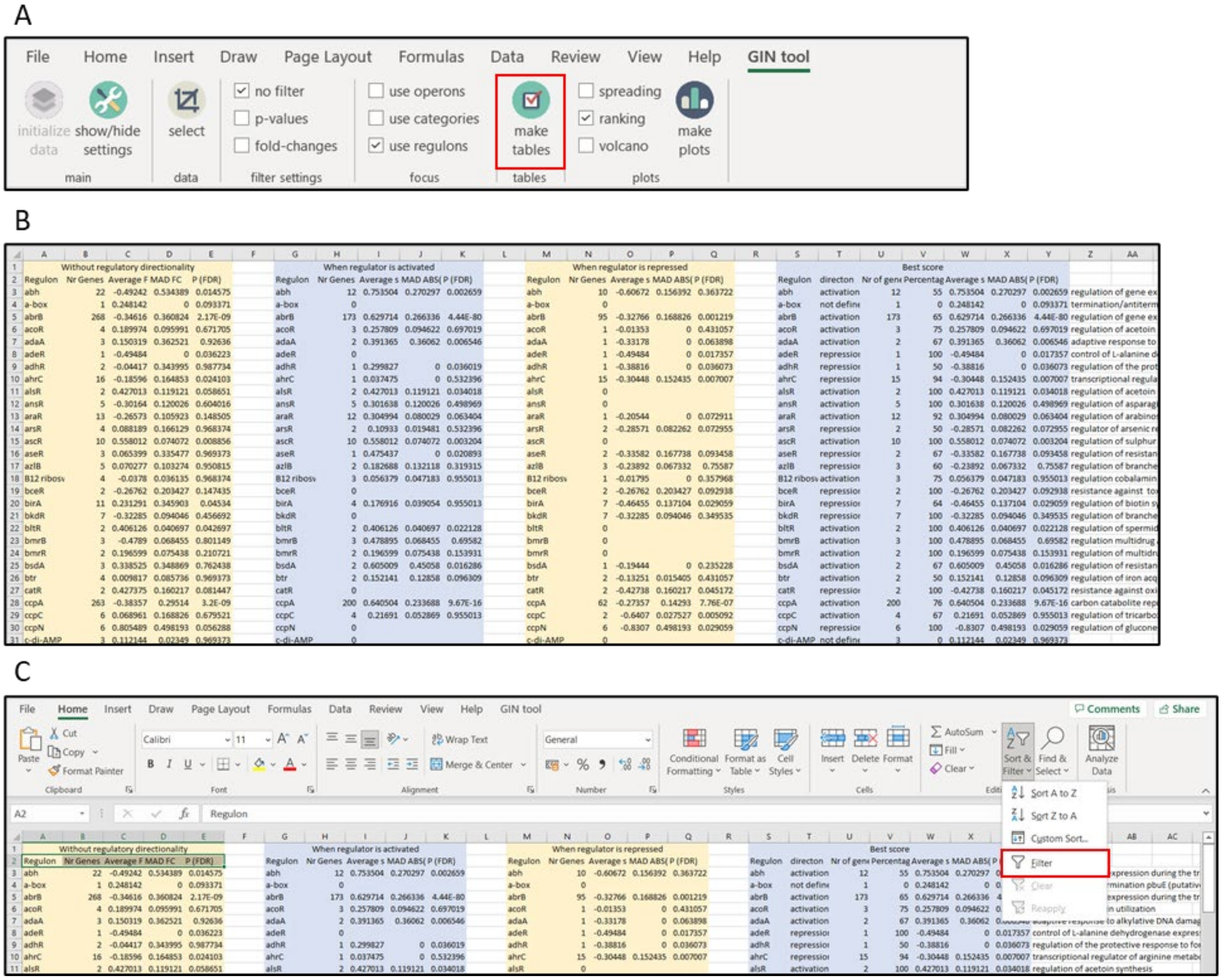

Figure 13: Creating tables with regulon information. A) Selecting the right options for creating tables; B) Sheet "Mapping_details"; C) Adding filters to headers to enable filtering and sorting of the first table.

6.4 Determination of most relevant regulon regulation

The problem with ranking of regulons is that the average fold-change calculation is based on all the genes of a regulon. However, a regulator might repress certain genes and activate others. This bifurcation will reduce the average fold-change. For example, when a regulator activates 5 genes and represses 5 genes then the average fold-change can be close to zero, whereas the regulator/regulon is activated. To correct for this, GINtool uses the regulation direction that has been defined when uploading the regulon file in the "up/down regulation mapping" window discussed in section 4.1 (Figure 4B). This information indicates whether a regulator activates or represses a gene. To incorporate this information, GINtool calculates the situation when the regulator is either activated or repressed, resulting in the second and third table in "Mapping details" that are labelled "When regulator is activated" and "When regulator is repressed" (Figure 13B). The fourth, most right, table called "Best score", lists the best scoring activity deducted from the second and third tables, based firstly on the number of genes that follow the same type of activity, and in case the numbers are the same, based on the activity with the highest average FC. This final table also indicates the fraction of genes that follow the most relevant regulon activity relative to all the genes that belong to a regulon, indicated in % in column V. This percentage is another important value that indicates how relevant a regulon is for explaining the transcriptome data (see also below).

It is possible that the mode of regulation of a gene has not been defined and therefore could not be selected in the "up/down regulon mapping" window. If this was the case, then the direction of regulon activity in table "Best score" will be 'not defined' (*e.g.* for regulon 'a-box' in row 4 in Figure 13B). Importantly, when the directionality of gene regulation cannot be defined, then this will also affect the fraction of genes that follow the most relevant regulon activity, and in that case the % in column V can be lower than 50%.

The result of the "Best score" table is automatically displayed as bubble plot when the GINtool option "make plots" is used and the tick box "ranking" is active (see section 6.2). This plot is called "RegRankPlotBest_v1" (Figure 14A). In this plot the negative or positive fold-change indicate whether a regulator is activated or repressed, respectively. *Importantly*(!), this can be a different direction than the measured fold-changes of genes, since an activated repressor (positive FC in the plot) will reduce the expression of genes. That is why in Figure 14A "melR" has a positive value whereas the average fold-change of the MelR regulon was negative as shown in the "RegRankPlot" bubble plot in Figure 11B.

The problem with "RegRankPlotBest_v1" is that it is unclear how many genes of a regulon form the bubble data points (columns H and N versus column B in the "Mapping_details" sheet). However, the "Best score" table also provides the fraction of the genes that were selected, % in column V, which provides an alternative way to display the most relevant regulons for a quick visual inspection by plotting the average fold-change of the best scoring activity against the fraction (%) of genes in a regulon that are responsible for the calculated fold-change. Such bubble plot is automatically made when the GINtool option "make plots" is used and the tick box "ranking" is on, and is called "RegRankPlotBest_v2" (Figure 14B). In the example, the bubble size has been reduced and the 50 % of the regulons with the highest p-values are not selected, thus not shown. Since only the most relevant activity of the regulons are chosen for the "Best score" table, the fractions are mostly above 50 %. However, as explained above, the % may be below 50 % when not all genes in a regulon have a defined mode of regulation. Finally, also for this graph type, bubble size and names can be adjusted using standard Excel tools, and the most relevant regulons can also be selected by setting cut-off values in the "filter settings" window as explained in 6.2.

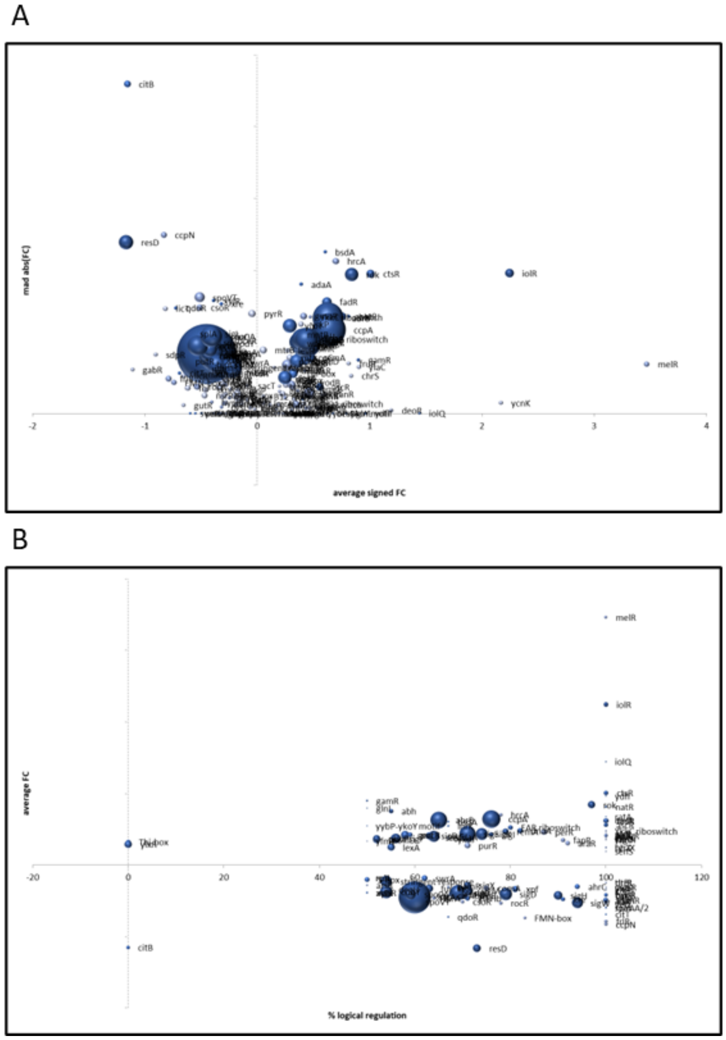

Figure 14: Bubble plots of most relevant regulon activities. A) “RegRankPlotBest_v1” shows the most relevant regulation of each regulon; B) “RegRankPlotBest_v2” shows the fraction of genes that follows the most relevant regulon regulation. 50 % regulons with highest p-values are filtered and bubbles are rescaled to 25.

It can be useful to have the “Best score” data of regulons available for every gene. This is possible by using the “make tables” function in GINtool. As described at the beginning of this section, this function produces two table sheets among which one is called “Mapped” (Figure 15).

The table indicates the details of a gene (ID, name, fold-change, p-value, function, description), the number of regulons (“Tot# Regulons”), and names of the regulons to which the gene belongs. In addition, it shows in between brackets the average fold-change (FC) and fraction (%) of genes of the most relevant state of a regulon, based on the “Best score” table in the table sheet “Mapping_details” (e.g. sigE(FC:0.44 71%)). Of note, regulons for which the mode of regulation (activation/repression) could not be indicated will show a 0%.

	A	B	C	D	E	F	G	H	I	
1	Gene id	Gene name	Fold-change	P-value	Function	Description	Tot# Regulons	Regulon_1	Regulon_2	Regulo
2	BSU_11549	yizD	2.002516245	0.096155044	unknown	unknown	1	sigE(FC:0.44 71%)		
3	BSU_21030	yonP	1.62902704	0.227919906	unknown	unknown	0			
4	BSU_14629	sr1	1.601961295	0.002606691	control of arginine m	small regulatory RNA	2	sigA(FC:-0.45 60%)	ccpN(FC:-0.83 100%)	
5	BSU_36720	ywmE	1.504901617	0.152470565	survival of ethanol st	general stress protein	1	sigB(FC:-0.39 69%)		
6	BSU_20300	yorP	1.504410801	0.181840906	unknown	unknown	0			
7	BSU_06319	ydzX	1.503871939	0.177236706	unknown	unknown	0			
8	BSU_27809	yrzT	1.476434051	0.218651856	unknown	unknown	0			
9	BSU_06120	ydjB	1.440394618	0.039960867	unknown	unknown	0			
10	BSU_20620	yoqI	1.409932161	0.183178746	unknown	unknown	0			
11	BSU_32090	yuiA	1.395148599	0.019006795	unknown	unknown	2	codY(FC:-0.38 54%)	tnrA(FC:0.30 57%)	
12	BSU_07190	yezD	1.392904273	0.180524672	unknown	unknown	1	cymR(FC:-0.43 70%)		
13	BSU_19430	cdaS	1.357653606	0.145220924	synthesis of c-di-AMP	sporulation-specific d	1	sigG(FC:0.40 64%)		
14	BSU_29020	gapB	1.35051979	0.002368569	anabolic enzyme in gl	glyceraldehyde-3-phc	2	ccpN(FC:-0.83 100%)	sigA(FC:-0.45 60%)	
15	BSU_14720	ylaB	1.339800452	0.206763112	unknown	unknown	3	spx(FC:-0.43 62%)	sigA(FC:-0.45 60%)	ylaC(F
16	BSU_02180	ybfE	1.334650537	0.051212434	unknown	unknown	0			
17	BSU_26980	yraE	1.321525618	0.257068695	unknown	forespore-specific sp	1	sigG(FC:0.40 64%)		
18	BSU_16799	ylzJ	1.265454223	0.236555726	control of TepA activi	germination protein,	2	spoVT(FC:-0.51 60%)	sigG(FC:0.40 64%)	
19	BSU_23270	ribE	1.264654964	0.002935057	riboflavin biosynthesi	riboflavin synthase (a	2	sigA(FC:-0.45 60%)	FMN-box(FC:-0.74 83%)	
20	BSU_36680	ywmF	1.231746679	0.169942124	unknown	unknown	1	sigB(FC:-0.39 69%)		
21	BSU_misc_RNA_59	BSU_misc_RNA_59	1.211348333	0.192298838			0			
22	BSU_28260	leuC	1.20879374	0.000917827	biosynthesis of leucin	3-isopropylmalate de	4	sigA(FC:-0.45 60%)	tnrA(FC:0.30 57%)	codY(F
23	BSU_22849	ypzH	1.157034029	0.301486735	unknown	unknown	0			
24	BSU_11290	yjaV	1.115583996	0.241513723	[[category|Sporulatic	[[protein|SigE]]-depe	1	sigE(FC:0.44 71%)		
25	BSU_18899	yozX	1.099514197	0.317003651	unknown	unknown	0			
26	BSU_26380	yqaB	1.098258018	0.214665205	unknown	similar to phage-relat	0			

Figure 15: Overview of the table in the sheet named "Mapped". Regulon information is shown per gene.

6.5 Volcano plot display

A popular way to display transcriptome data is by means of a Volcano plot where the fold change (X-axis) of genes is plotted against the p-value (Y-axis). GINtool includes an option to create a volcano plot based on regulon information. Choose "volcano" in the "plot" tab and press "make plots" (Figure 16A, red box). The "Select categories/regulons" window pops up. Select the relevant regulons or categories (see above) and press "Ok". Figure 16B shows the resulting volcano plot for regulons. In this case, the colors are related to the Best Score %, so that the most relevant regulons can be displayed. GINTool divides regulons in five colors. In Figure 16B, the colors blue, yellow, grey and orange correspond to regulons with a relevance % between 100-90 %, 90-80 %, 80-75 % and 75-67 %, respectively. Regulons with relevance of <67 % can also be displayed, but have been unselected in Figure 16B. The relevance % is based on the "best score" table described in section 6.4.

It is possible to trim the y-axis to zoom into the datapoints closer to the x-axis. This can be done in Excel by e.g. changing the y-axis to a log scale. However, GINtool also provides the option to set a maximum y-axis value, by checking "maximize extremes" in the "Select categories/regulons" window and fill in the value.

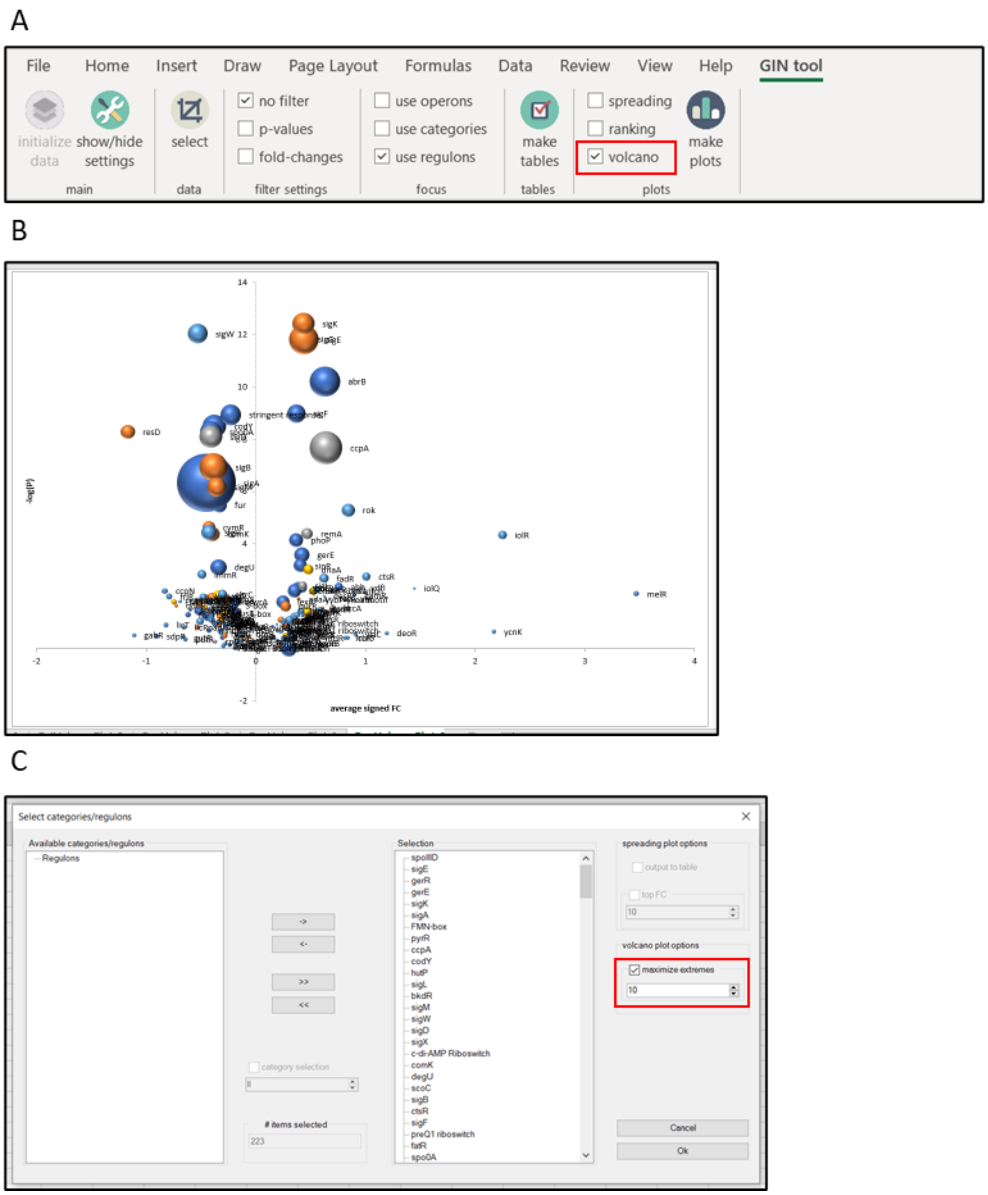

Figure 16. Volcano plots. A) Selecting the right options for creating volcano plots; B) Volcano plot of regulons. The colors blue, yellow, grey and orange correspond to regulons of which 100-90 %, 90-80 %, 80-75 % and 75-67 % of their genes, respectively, show a differential expression corresponding to the same regulator activity; C) Selecting a maximum value for the y-axis of a volcano plot.

7. Analysis of functional categories

*First follow the steps described in **section 4**.*

<u>7.1 Visual analysis of functional categories using spread plots</u>

GINtool gives the option to rank functional categories based on the average fold-change of all genes in a functional category. The distribution of fold-changes of genes of functional categories can be visualized in

a spread plot. Start by ticking “use categories” as “focus” and “spreading” for “plots” (Figure 17A). Click “make plots”, the “Select categories/regulons” window will pop up (Figure 17B).

By clicking on “Categories” in the left window “Available categories/regulons”, the subcategories become visible. Categories and subcategories can be selected and moved to the right window “Selection” by clicking on the single arrow button. It is also possible to select different levels of categories by ticking the “category selection” box, indicating the level you would like to display and subsequently clicking the double arrow button (Figure 17B, red box). The most detailed categories are levels III and IV for the *B. subtilis* annotation. To produce the spread plot, click “Ok”. This will create a new sheet called “CatSpreadPlot”, displaying the fold-changes of all genes in the selected categories, whereby the categories are ranked based on the average fold-change (Figure 6C). The x-axis indicates the fold-change.

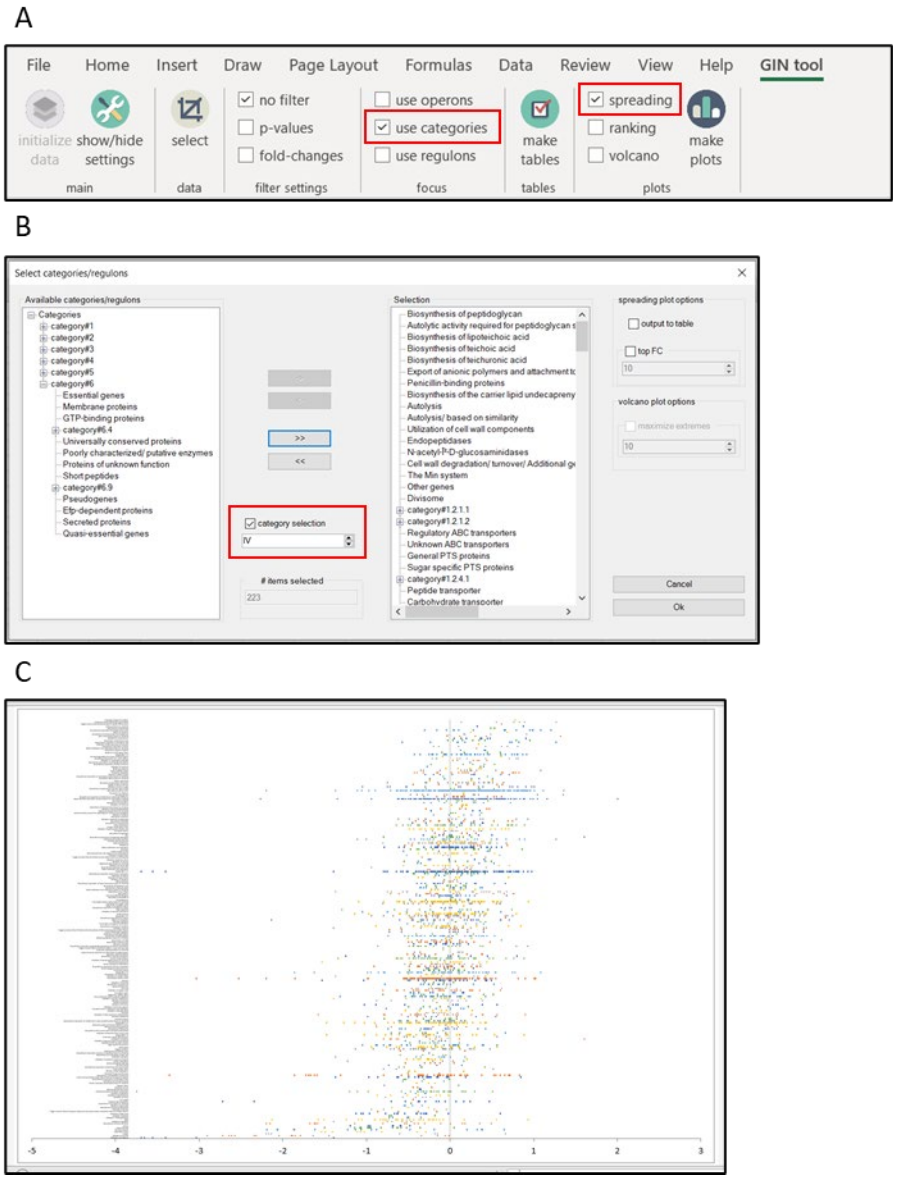

Figure 17: Creating a spread plot of functional categories. A) Selection of focus and type of plot; B) Selection of categories of interest; C) Spread plot “CatSpreadPlot” with information of fold-changes of all genes in the selected

categories, in descending order.

Often only the most up- or down-regulated categories are interesting. To show these, go back to "make plots" and tick the "top FC" (fold-change) box and set the number of most strongly regulated categories to be displayed, *e.g.* 20 (Figure 18A, red box). By clicking on "Ok" a new sheet is created called "CatSpreadPlotTop20FC", displaying the 20 most affected selected categories (Figure 18B).

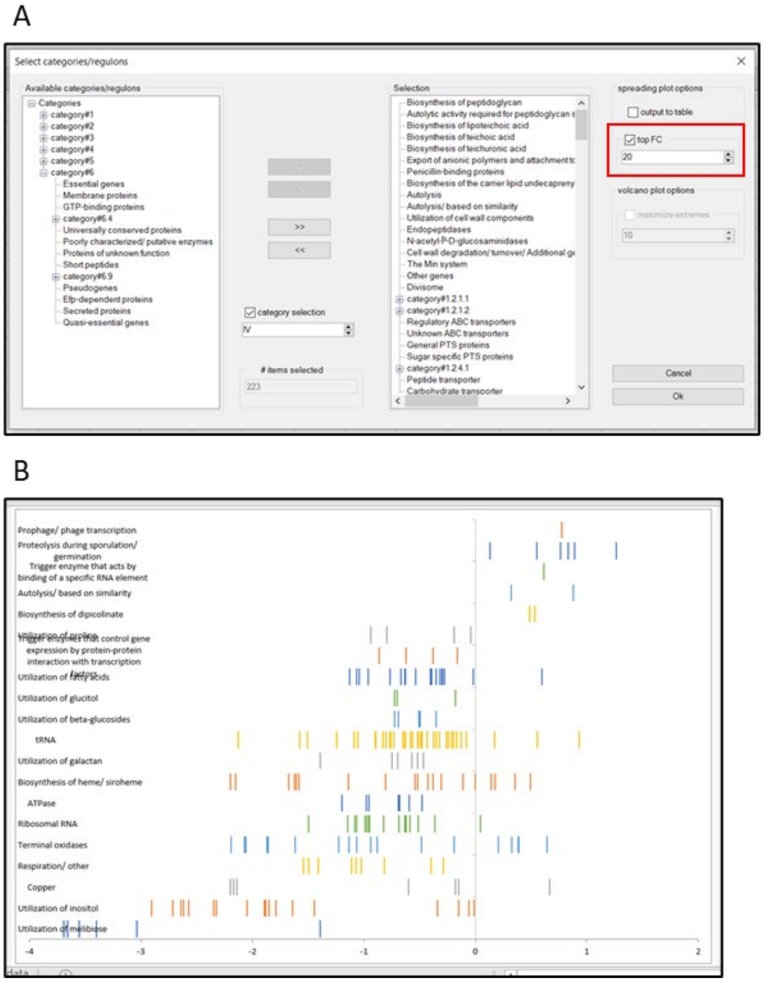

Figure 18: Creating a spread plot of top 20 affected categories based on average fold-changes. A) Selection of spreading plot options; B) Plot of top 20 most affected selected categories based on average FC "CatSpreadPlotTop20FC".

As default, the most upregulated categories are displayed on top. This order can be reversed. For this, click on the "show/hide settings" tab so that the "sort direction" window becomes visible (Figure 19A, red box). Tick "ascending", click on "show/hide settings" and "make plots", select the categories of interest, if necessary also select the best scoring categories using the FC or p-value selection, and click on "Ok". Figure 19B shows the reversed result of Figure 17C.

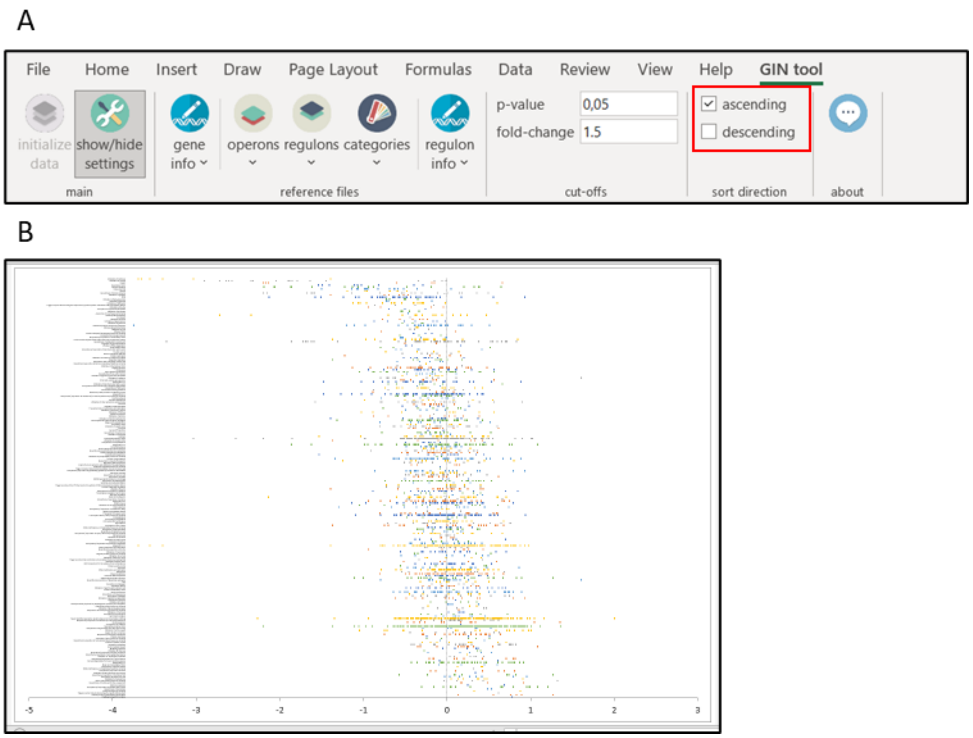

Figure 19: Creating a spread plot of all selected categories in ascending order. A) In settings bar, tick direction in "sort direction"; B) Spread plot "CatSpreadPlot" with information of fold-changes of all genes in the selected categories, in ascending order.

It can be useful to know what genes are part of the selected categories and what their fold-change and p-values are. To obtain this information click again on "make plots" but this time tick the "output to table" box (Figure 20A, red box). In the example the 10 strongest up- or down regulated categories have been selected. After clicking "Ok" a sheet called "CatSpreadTabTop10FC" is created, listing the selected categories with the genes that are part of the category and the related fold-change (FC) (Figure 20B). In addition, a sheet with the corresponding spread plot called "CatSpreadPlotTop10FC" is created.

A

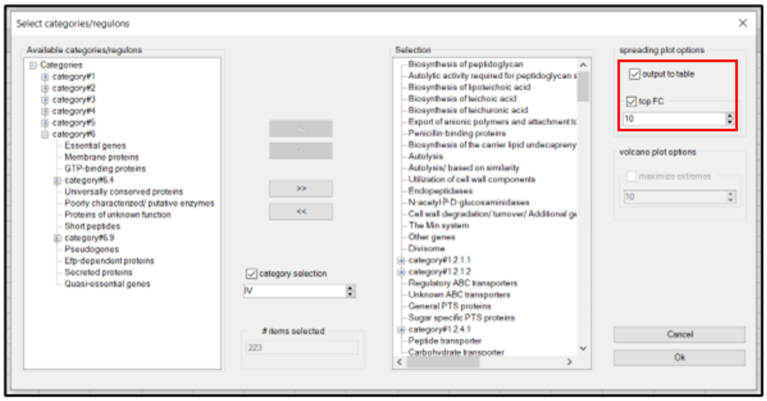

B

	A	B	C	D
1	Category	Gene	FC	p-value
2	Prophage/	BSU_2104	0.773726	0.174202
3	Proteolysi:	BSU_1679	1.265454	0.236556
4	Proteolysi:	BSU_0043	0.891788	0.274411
5	Proteolysi:	BSU_2554	0.831163	0.250504
6	Proteolysi:	BSU_2797	0.765673	0.323642
7	Proteolysi:	BSU_1679	0.553084	0.387679
8	Proteolysi:	BSU_1531	0.134103	0.827218
9	ATPase	BSU_3682	-0.47647	0.109408
10	ATPase	BSU_3684	-0.59033	0.062258
11	ATPase	BSU_3681	-0.59278	0.044574
12	ATPase	BSU_3683	-0.68075	0.030283
13	ATPase	BSU_3680	-0.68616	0.027126
14	ATPase	BSU_3685	-0.6901	0.03332
15	ATPase	BSU_3686	-0.95737	0.00537
16	ATPase	BSU_3687	-0.97862	0.002724
17	ATPase	BSU_3688	-1.196	0.000577
[illegible]	[illegible]	[illegible]	[illegible]	[illegible]

Figure 20: Generation of table with fold-change and p-value of genes in selected categories. A) Selection of settings in "Select categories/regulons"; B) Part of the table in sheet "CatSpreadTabTop10FC".

An alternative way to select categories is by specifying cut-off values for either the fold-change or p-value of the genes that are included in the analysis. Click "show/hide settings" to open the settings bar. The desired cut-offs can be determined in the cut-offs window (Figure 21A, red box). By default, no cut-offs are activated. To use a cut-off, click "show/hide settings" and tick "fold-changes" in the "filter settings" window in the analysis bar (Figure 21B, red box). The plots and tables can be created as described above. For example, the FC cut-off can be changed to 2. After filtering the genes based on a fold-change cut-off of 2, only 14 categories remain (Figure 21C). The same procedure can be followed to select genes based on p-values, or based on both cut-offs, by ticking the appropriate boxes in "filter settings" (Figure 21B).

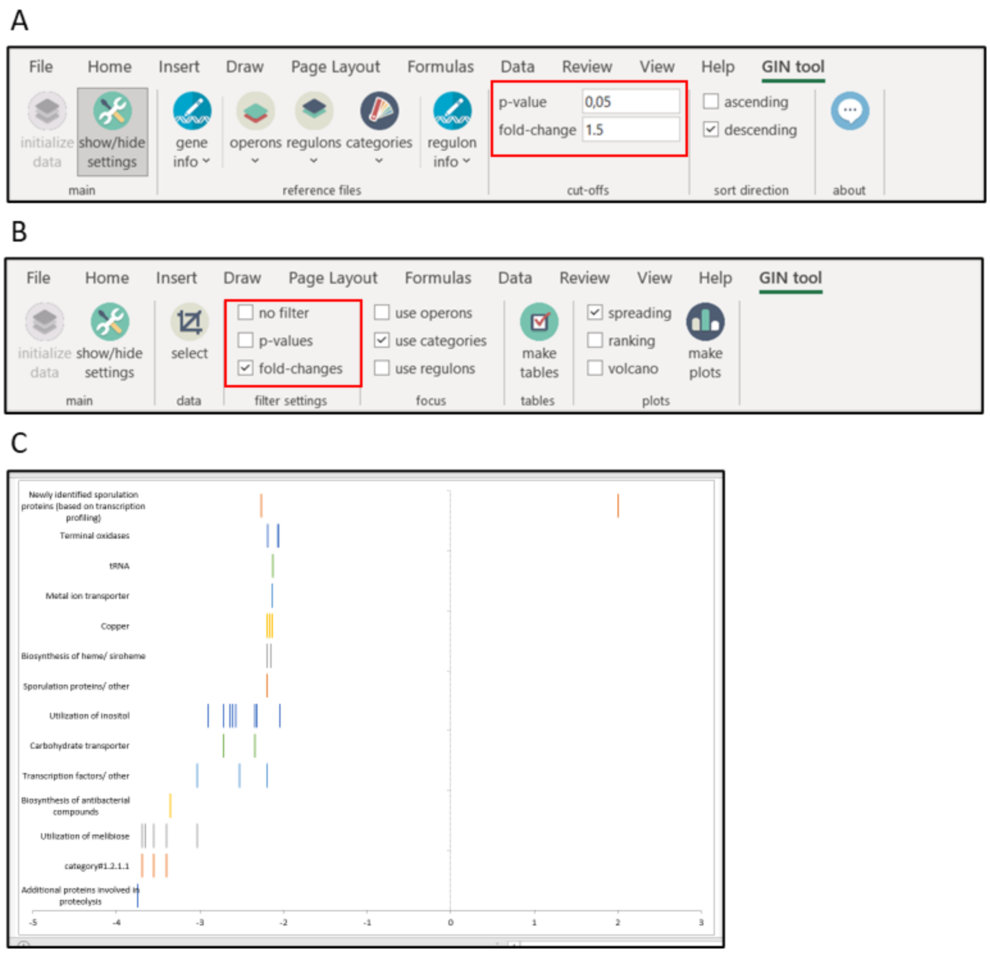

Figure 21: Creating a spread plots of categories after filtering genes. A) Cut-off values can be specified in the "cut-offs" window in the settings bar; B) Cut-offs can be activated by ticking the appropriate boxes in "filter settings" in the analysis bar; C) Example of "CatSpreadPlot" after filtering genes below a fold-change of 2.

! If the fold-changes in the data set are log2 transformed, then the FC cut-off is also interpreted as such.

! The fold-change cut-offs are absolute values, *i.e.* fold 2 can be both 2-fold downregulation and 2-fold upregulation.

7.2 Visual analysis of functional categories using bubble plots

The large number of categories can make it difficult to visually determine how relevant they are. To facilitate this, GINtool can make bubble plots based on the average fold-change of the genes in a category (x-axis) and the spreading of fold-changes of genes, defined as the mean absolute deviation (MAD) of the fold-changes (y-axis). Categories that show a robust regulation should have fold-changes that are close together, resulting in lower MAD values.

To make these bubble plots, tick "use categories" in the "focus" window and "ranking" in the "plots" window (Figure 22A, red boxes). Click "make plots" and the "select categories/regulons" window will pop up. Select the relevant categories as described above (section 7.1). Of note, filters for genes can be

activated (Figure 21A). In this example, no filters are active and the IVth category level is selected.

After selecting categories and clicking "Ok", four sheets are created: "CatRankPlot", "CatRankPlotBest_v1", "CatRankPlotBest_v2" and "CatRankTable". "CatRankTable" is the same table as "Mapping_details" which will be discussed in section 7.3, "CatRankPlotBest_v1" and "CatRankPlotBest_v2" will be discussed in section 7.4. An example of "CatRankPlot" is shown in Figure 22B.

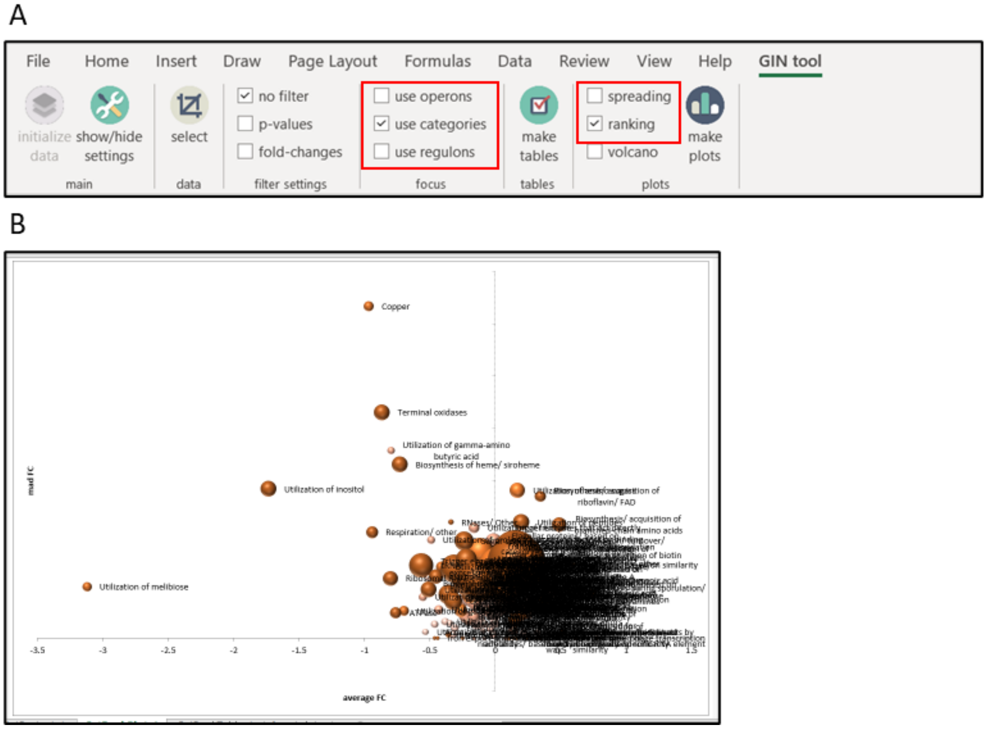

Figure 22: Creating a bubble plot of the average regulation of all selected categories. A) Selection of correct settings for creating bubble plots of categories; B) Example bubble plot "CatRankPlot" of all included categories and genes. Color intensity corresponds to ranking in average p-values. Bubble size corresponds to the number of genes in a category.

The use of a bubble plot provides the possibility to also display the size of categories, *i.e.* the number of genes of a category. The example in Figure 22B displays bubbles with different color intensities. The color intensity is related to the average p-value of the genes in the category.

The categories are divided into 5 fractions based on average p-values: the 10 % categories with the lowest p-values, then the 10-20 %, 20-33 and % 33-50 % fractions, and finally a fraction containing half of all categories (50 %) with the highest p-values. This classification enables the removal of categories with high average p-values by using the Excel "Chart filter" tool that is activated by clicking on the graph, as shown in Figure 23. The scale of the bubble size can be adjusted using the "Format data series" tool that becomes available by right-clicking on a bubble (Figure 23B). In this example, the scale is reduced to 25. Of course, the plots can be converted and adjusted using other standard Excel tools, and the graphs can be exported for furter editing, which can be useful to reposition the names of bubbles or change the colors.

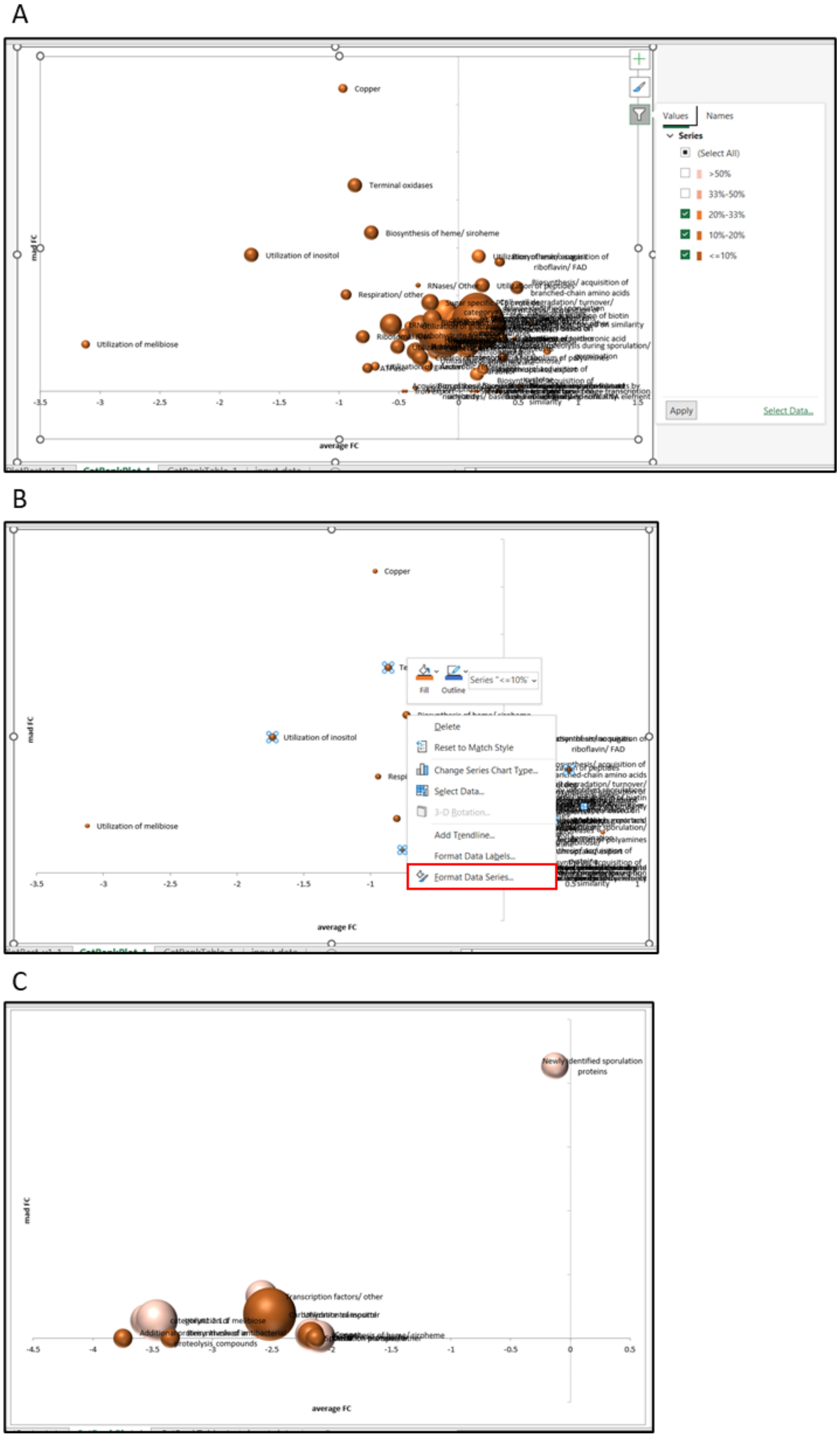

Figure 23: Creating bubble plots of categories based on preferences. A) Selecting bubbles based on average p-value of genes in a category; B) Rescaling bubble sizes; C) Bubble plot of categories after filtering genes with FC cut-off of 2.

It is possible to display the bubble plots of categories after filtering the genes in the categories based on fold-change and p-value (Section 7.1, Figure 21A). When a fold-change cut-off of 2 is active, fewer categories remain and distribution of colors is now based on the ranking of these 15 regulons (Figure 23C).

7.3 Functional category evaluation using tables

The average fold-change, MAD- and p-values can also be listed in a table. To do this, click on the "make tables" tab in the "tables" window (Figure 24A, red box). This creates two sheets: "Mapping details" and "Mapped". "Mapped" will be discussed in section 7.4. The "Mapping details" sheet contains four different tables (Figure 24B). The first table "Plot data" shows the number of genes, average fold-change, MAD fold-change and average p-value for each regulon. The table enables a quick ranking according to any of these parameters, by selecting the headers and using the Excel option "Filter" in the "Sort & Filter" tab in the Home tab (Figure 24C, red box). Of note, when making bubble plots there is also a table created "CatRankTable". This table is the same as the "Mapping details" table, but then showing only the categories that were selected.

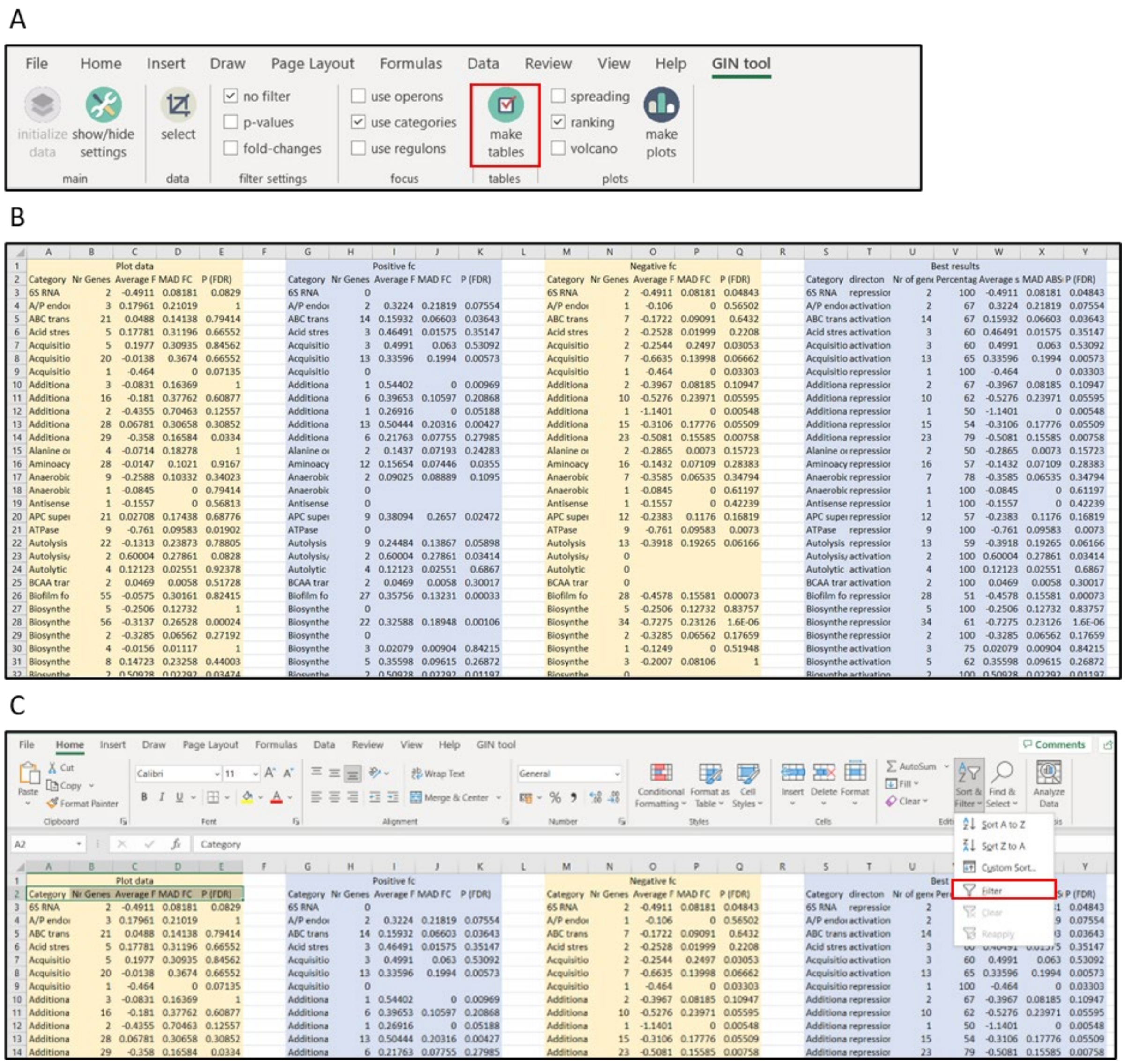

Figure 24: Creating tables with functional category information. A) Selecting the right options for creating tables; B) Sheet "Mapping_details"; C) Adding filters to headers to enable filtering and sorting of the first table.

7.4 Determination of most relevant functional category regulation

The problem with ranking of categories is that the average fold-change calculation is based on all the genes of a category. However, it is possible that some genes are activated whereas others are repressed. If this heterogeneity is the case, then the related category could be less relevant. To identify categories that show the most consistent activation or repression, GINtool can calculate the number of genes of a category that corresponds to either activation or repression of the category. This is automatically performed when "Mapping_details" is created (see section 7.3). This is reflected in the second and third table in "Mapping_details" that are labelled "Positive Fc" and "Negative Fc". The most right table called "Best results", lists the best scoring activity of the categories from the second and third tables, based firstly on the number of genes that follow the same type of activity, and in case the numbers are the same, based

on the activity with the highest average FC. This final table also indicates the fraction of category genes that follow the most relevant activity, indicated in % in column V.

The result of the "Best results" table is automatically displayed as a bubble plot when the GINtool option "make_plots" is used and the tick box "ranking" is active (see section 7.2). This plot is called "CatRankPlotBest_v1" (Figure 25A).

The problem with "CatRankPlotBest_v1" is that it is unclear how many genes of a category form the bubble data points (columns H and N versus column B in the "Mapping_details" sheet). However, the fourth "Best results" table also provides the fraction of the genes that were selected, % in column V, which provides an alternative way to display the most relevant categories for a quick visual inspection by plotting the average fold-change of the best scoring activity against the fraction (%) of genes in a category that are responsible for the calculated fold-change. Such a bubble plot is automatically made when the GINtool option "make plots" is used and the tick box "ranking" is on, and is called "CatRankPlotBest_v2" (Figure 25B). In the example, the bubble size has been reduced and the 67 % categories with the highest average p-values are not shown. Since only the most relevant activity of the categories are chosen, the fraction is always above 50 %. Finally, also for this graph type, bubble size and names can be adjusted using standard Excel tools, and the most relevant categories can also be selected by setting cut-off values in the "filter settings" window as explained in 7.2.

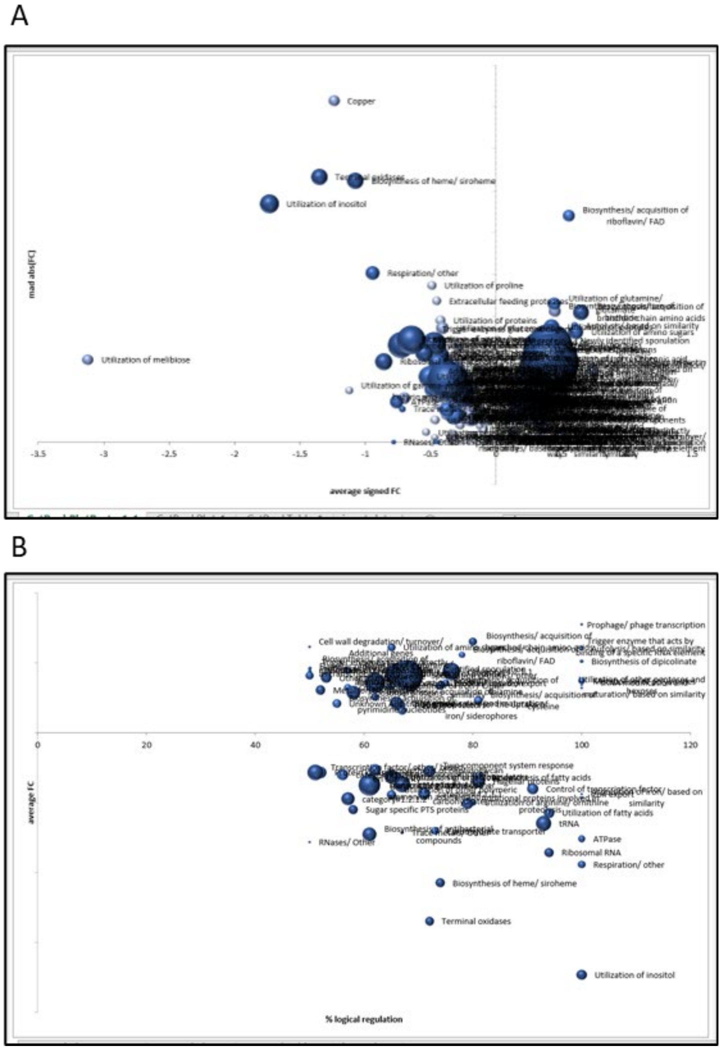

Figure 25: Bubble plots of most relevant category activities. A) "CatRankPlotBest_v1" shows the most relevant regulation of each selected category; B) "CatRankPlotBest_v2" shows the fraction of genes that follows the most relevant category regulation. 67 % categories with highest p-values are filtered and bubbles are rescaled to 25.

It can be useful to have the "Best results" data of categories available for every gene. This is possible by using the "make tables" function in GINtool. As described at the beginning of this section, this function produces two table sheets among which one called "Mapped" (Figure 26).

The table indicates the details of a gene (ID, name, fold-change, p-value, function, description), the number of categories ("Tot# Categories"), and names of the categories to which the gene belongs. In addition, it shows in between brackets the average fold-change (FC) and fraction (%) of genes of the most relevant state of a category based on the "Best results" table in the table sheet "Mapping_details" (e.g. SP-beta prophage (FC:0.48 54%)).

	A	B	C	D	E	F	G	H	I	Cat
1	Gene id	Gene name	Fold-change	P-value	Function	Description	Tot# Categories	Category_1	Category_2	Cat
2	BSU_11549	yizD	2.002516245	0.096155044	unknown	unknown	2	Newly identified spor	Membrane proteins(FC:-0	
3	BSU_21030	yonP	1.62902704	0.227919906	unknown	unknown	1	SP-beta prophage(FC:0.48 54%)		
4	BSU_14629	sr1	1.601961295	0.002606691	control of arginine m	small regulatory RNA	3	Biosynthesis/ acquisit	Utilization of arginine	Reg
5	BSU_36720	ywmE	1.504901617	0.152470565	survival of ethanol st	general stress protein	2	General stress protei	Membrane proteins(FC:-0	
6	BSU_20300	yorP	1.504410801	0.181840906	unknown	unknown	1	SP-beta prophage(FC:0.48 54%)		
7	BSU_06319	ydzX	1.503871939	0.177236706	unknown	unknown	1	Proteins of unknown function(FC:-0.35 60%)		
8	BSU_27809	yrzT	1.476434051	0.218651856	unknown	unknown	1	Proteins of unknown function(FC:-0.35 60%)		
9	BSU_06120	ydjB	1.440394618	0.039960867	unknown	unknown	2	Prophage 3(FC:-0.30 (	Proteins of unknown func	
10	BSU_20620	yoqI	1.409932161	0.183178746	unknown	unknown	1	SP-beta prophage(FC:0.48 54%)		
11	BSU_32090	yuiA	1.395148599	0.019006795	unknown	unknown	1	Proteins of unknown function(FC:-0.35 60%)		
12	BSU_07190	yezD	1.392904273	0.180524672	unknown	unknown	1	Proteins of unknown function(FC:-0.35 60%)		
13	BSU_19430	cdaS	1.357653606	0.145220924	synthesis of c-di-AMP	sporulation-specific d	2	Metabolism of signall	Sporulation proteins/ oth	
14	BSU_29020	gapB	1.35051979	0.002368569	anabolic enzyme in gl	glyceraldehyde-3-pho	1	Gluconeogenesis(FC:0.58 50%)		
15	BSU_14720	ylaB	1.339800452	0.206763112	unknown	unknown	2	Membrane proteins(F	Proteins of unknown func	
16	BSU_02180	ybfE	1.334650537	0.051212434	unknown	unknown	2	Membrane proteins(F	Proteins of unknown func	
17	BSU_26980	yraE	1.321525618	0.257068695	unknown	forespore-specific sp	1	Spore coat protein/ based on similarity(FC:0.54		
18	BSU_16799	ylzJ	1.265454223	0.236555726	control of TepA activi	germination protein,	2	Proteolysis during spc	Additional germination pr	
19	BSU_23270	ribE	1.264654964	0.002935057	riboflavin biosynthesi	riboflavin synthase (a	1	Biosynthesis/ acquisition of riboflavin/ FAD(FC:		
20	BSU_36680	ywmF	1.231746679	0.169942124	unknown	unknown	2	General stress protei	Membrane proteins(FC:-0	
21	BSU_misc_RNA_59	BSU_misc_RNA_59	1.211348333	0.192298838			0			
22	BSU_28260	leuC	1.20879374	0.000917827	biosynthesis of leucin	3-isopropylmalate de	2	Biosynthesis/ acquisit	Phosphorylation on an Ar	
23	BSU_22849	ypzH	1.157034029	0.301486735	unknown	unknown	1	Newly identified sporulation proteins (based on		
24	BSU_11290	yjaV	1.115583996	0.241513723	[[category|Sporulatic	[[protein|SigE]]-depe	1	Sporulation proteins/ other(FC:0.41 68%)		
25	BSU_18899	yozX	1.099514197	0.317003651	unknown	unknown	1	Proteins of unknown function(FC:-0.35 60%)		
26	BSU_26380	yqaB	1.098258018	0.214665205	unknown	similar to phage-relat	1	Skin element(FC:0.45 62%)		

Figure 26: Overview of the table in sheet named “Mapped”. Category information is shown per gene.

7.5 Volcano plot display

Category values can also be automatically displayed as Volcano plots in GINtool, using the same description as for regulons in section 6.5. Fig. 27 shows an example. In this case all categories are displayed (relevance scores 100-90 %, 90-80 %, 80-75 %, 75-67 % and <67 %). Again, extreme p-values can be topped off by checking “maximize extremes” in the “Select categories/regulons”. However, in the example in Figure 27, the y-axis scale has been transformed to log-scale using the standard "Format Axis" options in Excel.

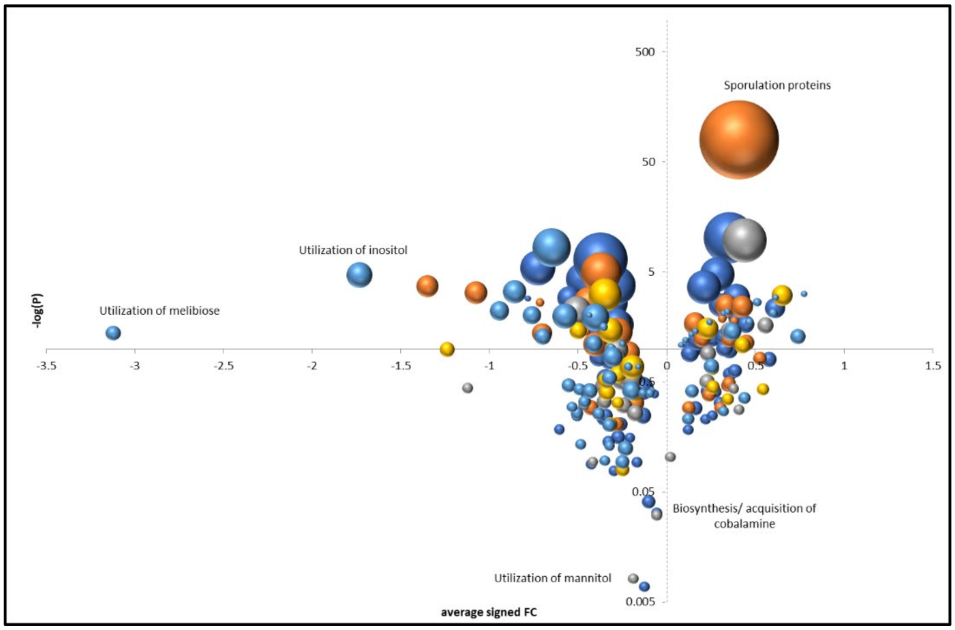

Figure 27. Volcano plot display of categories using a log-scale for the Y-axis. Colors indicate the relevance of regulation based on p-value sorting (100-90 %, 90-80 %, 80-75 %, 75-67 % and <67 %).

8. Overview of output sheets

8.1 Operons

Make tables:

- Mapped: for all genes, the fold-changes of the genes in its respective operon.

8.2 Regulons

Spread plots:

- RegSpreadPlot: plot of fold-changes of all included genes in the analyzed regulons.
- RegSpreadTab: table of fold-changes of all included genes in the analyzed regulons.
- RegSpreadPlotTopnFC: plot of fold-changes of all included genes in the top n regulons based on average FC.
- RegSpreakPlotTopnP: plot of fold-changes of all included genes in the top n regulons based on average p-value.
- RegSpreadTabTopnFC: table of fold-changes of all included genes in the top n regulons based on average FC.
- RegSpreadTabnP: table of fold-changes of all included genes in the top n regulons based on average p-value.

Bubble plots:

- RegRankPlot: bubbleplot of all selected regulons.
- RegRankPlotBest_v1: bubbleplot of most relevant regulon activity of all selected regulons.
- RegRankPlotBest_v2: bubbleplot of fraction of genes that follow most relevant regulon activity of all selected regulons.
- RegVolcanoPlot: Volcano plot displaying average p-values and fold-change of regulons. Categories are divided into 5 groups based on their Best score (relevance) %.
- RegRankTable: Mapping_details (see “make tables”) but only for the genes that are included in the analysis.

Tables:

- Mapped: overview of regulon information for each gene. For each regulon, the average fold-change of genes following most relevant regulon activity and fraction of genes that follow this activity are shown.
- Mapping_details: tables showing 1) regulon details without regulation directionality; 2) regulon details when regulator is repressed; 3) regulon details when regulator is activated; 4) best scoring regulon activity.

8.3 Categories

Spread plots:

- CatSpreadPlot: plot of fold-changes of all included genes in the analyzed categories.
- CatSpreadTab: table of fold-changes of all included genes in the analyzed categories.
- CatSpreadPlotTopnFC: plot of fold-changes of all included genes in the top n categories based on average FC.
- CatSpreakPlotTopnP: plot of fold-changes of all included genes in the top n categories based on average p-value.
- CatSpreadTabTopnFC: table of fold-changes of all included genes in the top n categories based on average FC.
- CatSpreadTabTopnP: table of fold-changes of all included genes in the top n categories based on average p-value.

Bubble plots:

- CatRankPlot: bubbleplot of all selected categories.
- CatRankPlotBest_v1: bubbleplot of most relevant category activities of all selected categories.
- CatRankPlotBest_v2: bubbleplot of fraction of genes that follow the most relevant category activity of all selected categories.
- CatVolcanoPlot: Volcano plot displaying average p-values and fold-change of categories. Categories are divided into 5 groups based on their Best score (relevance) %.
- CatRankTable: Mapping_details (see “make tables”) but only for the genes that are included in the analysis.

Tables:

- Mapped: overview of category information for each gene. For each category, the average fold-change of genes following the most relevant category activity and fraction of genes that follow this activity are shown.
- Mapping_details: tables showing 1) Category details without regulation; 2) Category details when genes are upregulated; 3) Category details when genes are downregulated; 4) best scoring Category activity.

9. Use of GINtool for transcriptome comparison

9.2 Comparing different experiments

The calculated average fold-change values for regulons in the “Mapped” sheet can be used as a convenient way to compare transcriptome data between different experiments by, e.g. using a scatter plot displaying the average fold-change value for a regulon from one experiment against the average fold-change found in the other experiment, as shown in Figure 28.

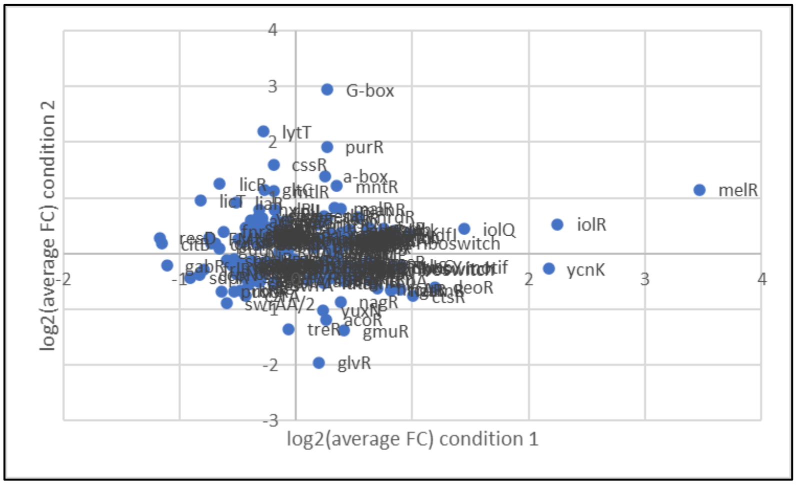

Figure 28: Scatter plot comparing average fold changes of regulons of two different experimental conditions. Datapoints are labelled with regulon names.

10. Calculation of MAD and average p-values

10.1 Median and Median absolute deviation (MAD)

As a robust measure, instead of the mean and standard deviation, the median and median absolute deviation (MAD) are used. The median is less prone to outliers and returns the 'middle' value of the observed values after sorting (from low to high). The MAD is a robust approximation of the spread around the median value and is calculated as the median of the residuals with respect to the median value determined earlier.

10.2 Average p-values

To give an estimate, expressed as p-value, of the gene sets, GINtool uses the fast gene-set enrichment analysis method as described by Subramanian, Tamayo et al. 2005 and Lachmann, Xie et al. 2022. The effect of grouping genes was taken into account by using the p-values adjusted for the False Discovery Rate.

Subramanian A, Tamayo P, et al. Gene set enrichment analysis: A knowledge-based approach for interpreting genome-wide expression profiles. PNAS. 2005;102: 15545–15550.

Lachmann A, Xie et al. blitzGSEA: efficient computation of gene set enrichment analysis through gamma distribution approximation. Bioinformatics. 2022;38: 2356–2357.

11. Making your own Bubble plot

GINtool makes bubble plots by dividing the data into 5 separate data series, i.e. those with an average p-value <0.0625, <0.125, <0.25, <0.5 or >=0.5. It can be useful to make your own bubble plots when you want to display a specific set of datapoints. Figure 29 shows a screenshot how to start. Firstly, one has to divide the data into separate data series. This enables adjusting e.g. the color of bubbles of a specific data series, or selectively displaying data series using the "Chart Filters" option. Data series require the

following information: Series name, Series X values, Series Y values and Series bubble size. In Figure 29 this is indicated in red in row 2 of the table. As you can see, the data points have been sorted into separate series based on Average p-values (column E). Next, insert a (Bubble) chart, right-klick on it and select "Insert Data" and "Add". Now you can select the first data series (Series name, Series X values, Series Y values, Series bubble size). After clicking "OK" you can add another data series, etc. To add the data labels indicated in Column A, right-mouse klick on a bubble, and klick "Add Data Labels". Then right-mouse klick again on a bubble or data label and select "Format Data Labels". This will open a window at the right side of the screen as shown in Figure 29. Select "Label Options" and tick the "Value From Cells" box and select the relevant data label series (relevant series in Column A). You have to do this for every data series.

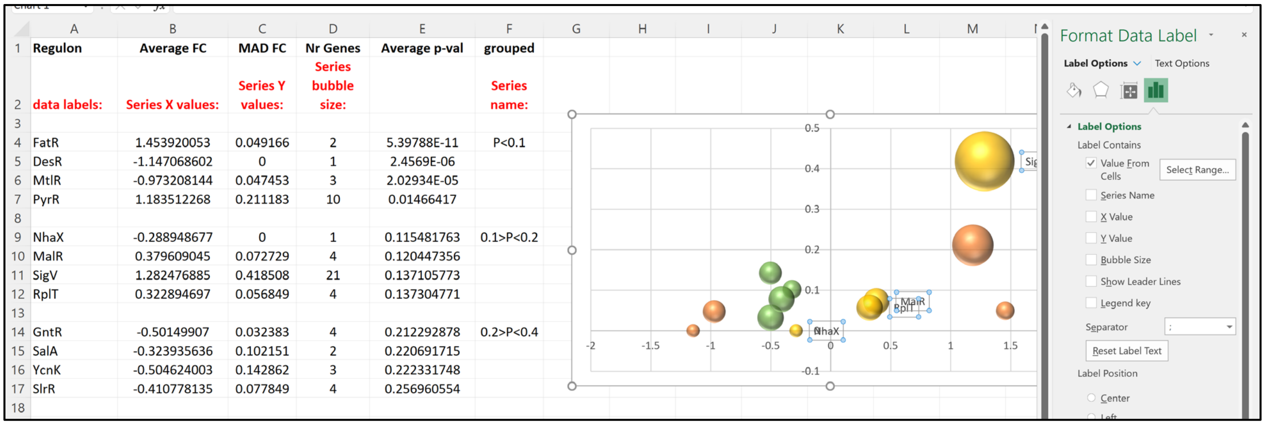

	A	B	C	D	E	F
1	Regulon	Average FC	MAD FC	Nr Genes	Average p-val	grouped
2	data labels:	Series X values:	Series Y values:	Series bubble size:		Series name:
3						
4	FatR	1.453920053	0.049166	2	5.39788E-11	P<0.1
5	DesR	-1.147068602	0	1	2.4569E-06	
6	MtlR	-0.973208144	0.047453	3	2.02934E-05	
7	PyrR	1.183512268	0.211183	10	0.01466417	
8						
9	NhaX	-0.288948677	0	1	0.115481763	0.1>P<0.2
10	MalR	0.379609045	0.072729	4	0.120447356	
11	SigV	1.282476885	0.418508	21	0.137105773	
12	RplT	0.322894697	0.056849	4	0.137304771	
13						
14	GntR	-0.50149907	0.032383	4	0.212292878	0.2>P<0.4
15	SalA	-0.323935636	0.102151	2	0.220691715	
16	YcnK	-0.504624003	0.142862	3	0.222331748	
17	SlrR	-0.410778135	0.077849	4	0.256960554	
18						

Figure 29: Example of a bubble plot in Excel.
