# Supplementary material for: Improving gene set enrichment analysis (GSEA) by using regulation directionality
Source: Microbiol Spectr. 2024 Jan 31;12(3):e03456-23. doi: 10.1128/spectrum.03456-23 (PMC10913524; doi:10.1128/spectrum.03456-23)
Supplement: Tables S1 and S2 — Strains, plasmids, and primer sequences. [file spectrum.03456-23-s0002.pdf]

Improving Gene Set Enrichment Analysis (GSEA) by using regulation directionality

Biwen Wang, Frans van der Kloet, Mariah B.M.J. Kes, Joen Luirink, Leendert W. Hamoen

Table S1. Strains and plasmids used in this study.

Strain	Genome type	Source
BSB1	*B. subtilis wildtype* 168 trp+;	Lab stock
BWB09	BSB1 trp+; Δ*xynA*, Δ*amyE*	This study
**Plasmid**		
pCS58	P_{amyQ}-*xynA, bleo(Km)*	Lab stock
pBW17	P_{amyQ}-*empty, bleo(Km)*	This study

Table S2. Primer sequences used in this study.

Name	Sequence (5'-3')	Target
BW05	CTAATTGAGAGAAGTTTCTATAGAATTTT	SpR-mazF-Fw
BW06	CTACCCAATCAGTACGTTAATTT	SpR-mazF-Rv
BW34	AAAGGAGCGATTTACATATGTAACAGATCATCCTTAATCA	pEmpty1-Fw
BW35	TGATTAAGGATGATCTGTTACATATGTAAATCGCTCCTTT	pEmpty1-Rv
BW41	CAGATCATCCTTAATCAGGGGTAGCTAACG	XnyADn-Fw
BW42	GAAACTTCTCTCAATTAGATTTCATGTAAACCGAGAACCA	XnyAEx-Rv
BW43	GTTATAACGCACCTTCCATT	XynAIn-Fw
BW44	AACTTTTTAACTAGAAAGCGCACGTAGTGTGATTATATCACCGTG	nyADn-Rv
BW45	GCAAAAGCCCTTATGAGGGCTTTTTTAATTGTTGTTTGCAGTAAC	XnyAUp-Fw
BW46	ACCCCTGATTAAGGATGATCTGATGTTACCTCCTATAATATTTTTTCCG	XnyAUp-Rv
BW47	AATTAACGTACTGATTGGGTAGTTCTTAGTTGGATTATCGGCAGC	XnyA-Fw
BW48	GGATGATCTGTTACCACACTGTTACGTTAGAACTTCCACTAC	XnyA-Rv
BW49	ATGATCAATTGGGGGCCGTTTTAACGATTGCTGCC	AmyEUp-Fw
BW50	TCCCGTCTAGCCTTGCCCTCTTGACACTCCTTATTTGA	AmyEUp-Rv
BW51	GGGCAAGGCTAGACGGGACTTACCGAAAGAAA	AmyEDn-Fw
BW52	TATAGAAACTTCTCTCAATTAGCCCGCTCTTTTGGCAGGCCGC	AmyEDn-Rv
BW53	AACGTACTGATTGGGTAGGCCATTCAGACATCTCCGA	AmyE-Fw
BW54	CAGACCTGGCATTGATCGTGCCTGTCAGTTTAC	AmyE-Rv
BW135	CTTTGAAGCTTGGCTGGTC	AmyEEx-Fw
ZT080	CACATTGTGAAATCTATTGACCGCAGTG	AmyEIn-Rv
